# Content Analysis of the Corporate Social Responsibility Practices of 9 Major Cannabis Companies in Canada and the US

**DOI:** 10.1001/jamanetworkopen.2022.28088

**Published:** 2022-08-23

**Authors:** Tanner Wakefield, Stanton A. Glantz, Dorie E. Apollonio

**Affiliations:** 1 Center for Tobacco Control Research and Education, School of Pharmacy, University of California, San Francisco; 2 Center for Tobacco Control Research and Education, University of California, San Francisco; 3 Department of Clinical Pharmacy, University of California, San Francisco

## Abstract

**Importance:**

The cannabis industry has sought to normalize itself and expand its markets in the 21st century. One strategy used by companies to generate positive public relations is corporate social responsibility (CSR). It is critical to understand these efforts to influence the public and politicians given the risks of increased cannabis use.

**Objectives:**

To analyze cannabis industry CSR behaviors, determine their characteristics, and compare their practices with those of the tobacco industry.

**Design, Setting, and Participants:**

This qualitative study of CSR activities conducted between January 1, 2012, and December 31, 2021, evaluated 9 of the 10 largest publicly traded cannabis companies in the US and Canada. Data were collected from August 1 to December 31, 2021. The 10th company was excluded because it engaged in cannabis-based pharmaceutical sales but not CSR. A systematic review of corporate websites and Nexis Uni was performed, resulting in collection of 153 news articles, press releases, and Web pages. Charitable and philanthropic actions were included. Themes were identified and interpreted using modified grounded theory.

**Main Outcomes and Measures:**

CSR activities and spending.

**Results:**

Nine major cannabis companies in the US and Canada engaged in CSR activities that encouraged increased consumption and targeted marginalized communities. Companies claimed these activities would mitigate the harms of cannabis prohibition, promote diversity, expand access to medical cannabis, and support charitable causes. They developed educational programs, sustainability initiatives, and voluntary marketing codes and used strategies similar to those used by tobacco companies to recruit public interest organizations as allies.

**Conclusions and Relevance:**

These findings suggest that cannabis companies developed CSR strategies comparable to those used by the tobacco industry to influence regulation, suggesting that cannabis companies should be included when addressing commercial determinants of health.

## Key Points

**Question:**

What are the corporate social responsibility (CSR) practices of the cannabis industry and how do they compare with tobacco industry practices?

**Findings:**

This qualitative study of 9 cannabis companies determined that they engaged in CSR activities nominally to mitigate the negative effects of cannabis prohibition; promoted diversity, equity, and inclusion; made charitable contributions; promoted cannabis medical utility and access; and addressed cannabis industry harms. Cannabis companies’ CSR strategies were similar to those used by tobacco companies to advance their interests and recruit third-party organizations as allies.

**Meaning:**

These findings suggest that these cannabis companies developed CSR initiatives similar to those used by the tobacco industry to influence politics and regulation, and this should be considered when evaluating commercial determinants of health.

## Teaser

This qualitative study reviews the corporate social responsibility practices of 9 cannabis companies in Canada and the US and assesses whether these activities are similar to those of tobacco companies.

## Introduction

As of February 2022, 19 states and Washington, DC, had voted to legalize recreational cannabis use in the US,^1^ although the US federal government classifies cannabis as a Schedule I substance with high abuse potential and no approved medical purpose^2^ despite therapeutic evidence.^3,4^ Cannabis companies (some represented on state regulatory boards)^5,6^ prefer policies likely to increase consumption.^5,7^ Canada legalized cannabis nationally in 2018,^8^ establishing a market based on advice from a task force including government, health, research, legal, law enforcement, and non–cannabis business representatives. Input was solicited from advocacy organizations and cannabis companies,^9^ the latter of which seek reduced taxes and regulations.^10^ Research on the effects of recreational cannabis legalization on cannabis use has been inconclusive, with studies reporting different increases and decreases in consumption after legalization.^11^ One study^12^ found that cannabis use increased in the past year and past month among populations of Asian, Hispanic, Native American, non-Hispanic White, and Pacific Islander race and ethnicity and among individuals reporting multiple races and ethnicities who were aged 21 to 30 years in states that had legalized medical and recreational cannabis. The study found no changes in cannabis use by non-Hispanic Black individuals or for any racial or ethnic demographic group aged 12 to 20 years.^12^ Another study of cannabis consumption in Canada found increased prevalence of cannabis use among middle-aged and older adults after recreational legalization.^13^ Federal surveys conducted in 2020 by their respective governments found that 17.9% of individuals 12 years or older in the US^14^ and 27% of persons 16 years or older in Canada consumed cannabis in the past 12 months.^15^ Further cannabis normalization could increase consumption as well as expand the reach and strength of companies selling cannabis products.

Corporate social responsibility (CSR) constitutes a company’s philanthropic, ethical, and economic activities beyond profit seeking,^16^ including mitigating environmental and societal impacts.^17^ Corporate social responsibility promotes brands and secures government goodwill that protects business interests.^18,19,20,21^ Controversial industries, including the tobacco industry, use CSR to bolster reputations burdened by *core stigma*,^22^ a term indicating that products, conduct, or customers have a negative social impact.^23^ Tobacco industry CSR activities ostensibly address harms caused by its products^24,25^ but blame consumers^26^ and present its programs as alternatives to regulation.^26,27^ That industry’s efforts include “youth smoking prevention” programs with forbidden fruit messaging,^28,29^ smokers’ rights groups that resist regulation,^30,31^ and pharmaceuticalization, which reoriented business toward nicotine replacement therapy.^32^ The World Health Organization Framework Convention on Tobacco Control,^33^ an international treaty adopted in 2003 to steer countries’ tobacco control programming, mitigate the global tobacco epidemic, and curb the tobacco industry’s interference in regulation, defines CSR as a form of advertising.^34^

As legal cannabis sales expand to new jurisdictions, the cannabis industry may seek to use CSR to gain legitimacy, secure approval, recruit allies, access government, and influence policy as it attempts to expand its customer base and legal markets. Past research on this question is limited: searches of academic databases found only 1 book chapter^35^ covering the CSR activities conducted by 2 cannabis companies operating in Colorado and 3 articles^36,37,38^ addressing CSR practices by cannabis companies, all of which stated that further research was needed. Comparison of cannabis companies’ CSR practices with those of the tobacco industry is useful because we can assess commonalities and differences between the practices of a nascent industry exiting illegality and a long-established sector with a product that has historically been legal.^39,40^ The recent legalization of cannabis in Canada and patchwork legalization of cannabis in 18 states in the US has led to the appearance of multiple smaller businesses alongside the multinational corporations in this sector. Owing to differences in the legality of cannabis within the US, cannabis businesses face varying market regulations. The prior criminalization of cannabis and resulting disparate racial and social harms has generated attention regarding how cannabis companies address diversity, equity, and inclusion, potentially influencing the focus of its CSR practices relative to the tobacco industry.

We reviewed cannabis CSR activities in the US and Canada between January 1, 2012, when Colorado first legalized recreational cannabis, and December 31, 2021. We sought to determine whether cannabis companies have CSR practices similar to those of the tobacco industry because both sell substances that are recreationally consumed and harmful to public health with comparable consumption modes (eg, smoking, vaping).

## Methods

For this qualitative study, we reviewed documents related to the CSR activities of cannabis companies between 2012 and 2021 using established methods from previous research of the tobacco,^41^ alcohol,^26^ and food^16^ industries. We sampled the 10 largest cannabis companies by market capitalization as of January 2021 identified by Nasdaq,^42^ an electronic securities trading market covering the US, Canada, and Europe.^43,44^ We excluded 1 pharmaceutical company making cannabis-derived treatments and not involved with recreational markets, leaving 9 companies^45,46,47,48,49,50,51,52,53,54,55,56,57,58,59,60,61,62^ (Table 1) in our sample.

**Table 1.** Overview of the Top 9 Multinational Cannabis Companies in January 2021 According to Nasdaq

Company	Market capitalization as of November 2021, $1 billion^a^	Headquarters (country) as of 2021	Year founded	Market sector
Curaleaf Holdings Inc	7.62^45^	Curaleaf Holdings Inc^46^ (US)	2010	Recreational and medical dispensaries, manufacturer
Innovative Industrial Properties	6.78 ^47^	Innovative Industrial Properties (US)^48,49^	2016	Real estate for medical cannabis operations
Canopy Growth Corporation	5.99^50^	Pitchbook (Canada)^51^	2013	Recreational dispensaries, manufacturer
Green Thumb Industries Inc	5.79^52^	Green Thumb Industries Inc (US)^53^	2014	Recreational dispensaries, manufacturer
Cresco Labs Inc	4.19^54^	Cresco Labs Inc (US)^55^	2013	Recreational and medical dispensaries, manufacturer
Trulieve	3.96^56^	Trulieve (US)^57^	2016	Medical dispensaries, manufacturer
Cronos Group Inc	2.25^58^	CNNMoney.com (Canada)^59^	2012	Manufacturer
GrowGeneration Corp	1.41^50^	GrowGeneration Corp (US)^60^	2008	Hydroponics supplier
Columbia Care	1.06^61^	United States Columbia Care (US)^62^	2012	Medical dispensaries

^a^ From Google Finance.

Our data collection, coding, analysis, and writing process followed the Standards for Reporting Qualitative Research (SRQR) reporting guideline. Our approach also relied on existing protocols for searching and analyzing industry documents.^63,64,65^ Because this study did not involve human subjects, informed consent was not applicable. This research was approved by the University of California, San Francisco, Institutional Review Board. Information regarding CSR activities was collected from August 1 through December 31, 2021, through systematic searches of cannabis company websites and Nexis Uni articles written in English. Websites were reviewed for CSR activities, including events, sponsorships, nonprofit partnerships, education initiatives, diversity, equity, and inclusion initiatives, donations, and sales drives.

Information on company websites was image captured and downloaded in PDF form. We searched Nexis Uni to find press releases and news coverage of cannabis companies’ CSR activities. Search terms included company names combined with the keywords *corporate social responsibility*, *social equity*, and *donate*. Subsequent snowball searches,^66^ a process in which key terms are identified in initial searches to find additional information, was used with brand and program names identified in initial searches. Philanthropic activities were included even if not labeled CSR. We identified 153 unique news articles, press releases, and Web pages that are described below.

We performed a content analysis to categorize evidence thematically, similar to studies used previously to analyze tobacco^67^ and pharmaceutical^68^ industry activities. One author (T.W.) with experience coding tobacco industry documents created a master file that summarized each cannabis company’s CSR activities provided in each document. Themes were developed inductively through iterative coding and categorization^69^ of language and informational patterns found in collected materials. Corporate social responsibility activities with language and foci designed to mitigate cannabis prohibition harms were grouped into a theme category, as were activities with language and foci regarding diversity, equity, and inclusion; charitable contributions; therapeutic and medical cannabis access promotion; and mitigation of cannabis industry harms.

We found that cannabis companies used CSR practices similarly to tobacco companies, claiming they voluntarily self-regulated by limiting youth access, making charitable contributions, and supporting advocacy organizations. We categorized activities under these classifications. When a document’s relevance or categorization was questioned, it was discussed by 2 authors (T.W. and D.E.A.) until agreement was reached.

## Results

The 9 companies in our sample operated in the US and Canada and were established between 2008 and 2016. Market capitalizations ranged from US $1.06 billion to US $7.62 billion in 2021 (Table 1). Two companies specialized in recreational cannabis manufacturing and retail sales, 2 were medical cannabis retailers (1 was also a manufacturer), 1 was a manufacturer, 2 manufactured and sold recreational and medical cannabis, 1 was a cannabis real estate company, and 1 supplied hydroponics. Two companies had ties to the alcohol and tobacco industries: Constellation Brands Inc, a US-based beer, wine, and spirits producer, purchased a 38.6% stake in Canopy Growth Corporation in 2017,^70^ and Altria Group Inc, the parent company of Philip Morris,^71^ purchased a 45% stake in Cronos Group Inc in 2019.^72^ We identified 5 cannabis CSR areas: (1) campaigns supposedly mitigating the harmful effects of past cannabis prohibition; (2) hiring initiatives characterized as promoting diversity, equity, and inclusion; (3) charitable contributions; (4) researching therapeutic cannabis uses and increasing medical access; and (5) efforts claiming to address cannabis legalization harms.

### Campaigns Companies Claimed Would Mitigate the Harmful Effects of Past Cannabis Prohibition

Seven companies claimed their CSR activities addressed harms from past cannabis prohibition (Table 2).^73,74,75,77,78,79,80,81,82,83,84,85,86,87,88,89,90,91,92,93,94,96,97,99,100^ Green Thumb Industries Inc^101,102,103,104,105^ and Cresco Labs Inc^91,92,95,106,107^ created business incubators and licensing assistance programs for members of racial and ethnic minority populations and communities harmed by cannabis prohibition. The License Education Assistance Program, launched by Green Thumb Industries Inc in 2019, reoriented programming to support 3 social equity license applicants in Illinois^102^ in August 2021.^103^ The Social Equity and Education Development business incubator developed by Cresco Labs Inc held 13 events between 2019 and 2020 that assisted 225 applicants pursuing Illinois retail licenses.^92^ Curaleaf Holdings Inc^73,74^ and Green Thumb Industries Inc^81^ funded nonprofits helping people with cannabis-related records rejoin society. Curaleaf Holdings Inc donated 10% of proceeds from BNoble cannabis product sales to 5 organizations helping formerly incarcerated people obtain work.^73^ Green Thumb Industries Inc allotted a portion of Good Green sales to a nonprofit grant program funding cannabis education, work training, employment assistance, and expungement services in communities harmed by cannabis criminalization.^82,83^ Green Thumb Industries Inc claimed it provided 3 Good Green grants as of 2021.^108^ Green Thumb Industries Inc collaborated with the Last Prisoner Project, a nonprofit founded by cannabis entrepreneurs^109,110^ dedicated to expunging sentences of formerly incarcerated people and reintegrating them.^81,111,112^ Three companies, Curaleaf Holdings Inc,^75,95,113^ Cresco Labs Inc,^91,114^ and Green Thumb Industries Inc,^82,83^ established quotas and initiatives to hire previously incarcerated people. Cresco Labs Inc^92,107^ and Green Thumb Industries Inc^90^ developed restorative justice CSR activities. Cresco Labs Inc established scholarship programs at 2 universities in Ohio for people or communities harmed by drug war policies.^95^ Green Thumb Industries Inc funded 4 scholarships in 2 Ohio schools (Olive-Harvey College and Cleveland School of Cannabis) for students seeking cannabis industry involvement.^84,115^ Canopy Growth Corporation,^79,80^ Curaleaf Holdings Inc,^113^ Green Thumb Industries Inc,^81,82,83,84^ Cresco Labs Inc,^91,92,93,107,114,116,117^ and Trulieve^97^ announced intentions to support efforts and organizations seeking to expunge criminal records.^99^ Six companies joined industry associations supporting restorative criminal justice: Trulieve and Cresco Labs Inc joined National Cannabis Roundtable, a US trade organization,^96^ whereas Canopy Growth Corporation, Curaleaf Holdings Inc, Cronos Group Inc, and Columbia Care joined the US Cannabis Council, a coalition seeking to end federal cannabis prohibition.^98,118^ Between 2019 and 2020, Cresco Labs Inc claimed it spent nearly $425 000 in staff hours and contributions toward restorative justice, including more than 90 hours of staffing at expungement events, sponsorship of 8 expungement events and 1 gun exchange, and assisting 22 restorative justice activations in California, Illinois, and Pennsylvania. Cresco Labs Inc reported assisting more than 1000 expungement seekers.^92,119^ Green Thumb Industries Inc contributed opening day proceeds from 4 store launches toward the Last Prisoner Project and Florida Rights Restoration Coalition,^86,87,88,89,120^ and Cresco Labs Inc collected donations at 1 store for the Last Prisoner Project.^94^

**Table 2.** Overview of CSR Activities Between 2012 and 2021 Addressing Cannabis Prohibition Harms of the 9 Largest Multinational Cannabis Companies as of January 2021

Company	CSR activity^a^										
	Expungement and funding	Scholarships and internships	Business incubators	Postincarceration assistance	Brands with dedicated funding to reform	Donated store opening proceeds to reform	Donated proceeds from wholesale to reform	Hiring initiatives	Job training	Programs with reform focus	Joined trade associations advocating reform
Curaleaf Holdings Inc	Curaleaf Holdings Inc,^73^ 2021	NA	NA	Curaleaf Holdings Inc, ^74^ 2021	Curaleaf Holdings Inc,^73,74^	NA	NA	Benzinga,^75^ 2021; PR Newswire,^76^ 2021	NA	Curaleaf Holdings Inc,^77^ 2021	MENAFN,^78^ 2021
Innovative Industrial Properties	NA	NA	NA	NA	NA	NA	NA	NA	NA	NA	NA
Canopy Growth Corporation	Canopy Growth Corporation,^79^ 2020; Ravilojan,^80^ 2020	NA	NA	NA	NA	NA	NA	NA	NA	NA	MENAFN,^78^ 2021
Green Thumb Industries Inc	GlobeNewswire,^81^ 2020; MENAFN,^82^ 2021; no author,^83^ 2021; GlobeNewswire,^84^ 2021; Green Thumb Industries,^85^ 2021	GlobeNewswire,^84^ 2021	Curaleaf Holdings Inc,^74^ 2021	GlobeNewswire,^84^ 2021; PR Newswire,^86^ 2021	MENAFN,^82^ 2021; no author,^83^ 2021	No author,^87^ 2020; GlobeNewswire,^88^ 2020; no author,^89^ 2020; PR Newswire,^86^ 2021	NA	NA	MENAFN,^82^ 2021; no author,^83^ 2021	Green Thumb,^90^ 2021	NA
Cresco Labs Inc	Business Wire,^91^ 2020; Cresco Labs Inc,^92^ 2021; Business Wire,^93^ 2021; Business Wire,^94^ 2019	No author,^95^ 2019	NA	NA	NA	NA	Business Wire,^93^ 2021	NA	NA	Cresco Labs Inc,^92^ 2021	MENAFN,^96^ 2020; MENAFN,^78^ 2021
Trulieve	Trulieve,^97^ 2021	NA	NA	NA	NA	NA	NA	NA	NA	NA	MENAFN,^96^ 2020
Cronos Group Inc	NA	NA	NA	NA	NA	NA	NA	NA	NA	NA	Business Wire,^98^ 2021; MENAFN,^78^ 2021
GrowGeneration Corp	NA	NA	NA	NA	NA	NA	NA	NA	NA	NA	NA
Columbia Care	NA	NA	NA	NA	NA	NA	NA	NA	NA	NA	MENAFN,^78^ 2021

Abbreviations: CSR, corporate social responsibility; MENAFN, Middle East and North Africa Financial Network; NA, not applicable.

^a^ Citations link to company involvement in CSR activity.

### Hiring Initiatives Characterized as Promoting Diversity, Equity, and Inclusion

Cannabis companies developed initiatives nominally promoting diversity, equity, and inclusion. These initiatives involved diversifying cannabis industry employment and promoting special populations (Table 3).^77,80,90,91,96,97,101,104,121,122,123,124,125,126,127,128,129,130,131,132,134,135,136,137,138,139,140,141,142^ Both activities expanded industry involvement in communities through hiring, goodwill, and creating local retailers. Five companies reported internal diversity, equity, and inclusion efforts. Canopy Growth Corporation planned to create diversity benchmarks and report its progress^80^; we were unable to verify whether they implemented those plans. Curaleaf Holdings Inc created internal employee resource groups for minority, LGBTQ+ (lesbian, gay, bisexual, transexual, and queer plus), parent, and female employees^143^ and established a diversity, equity, and inclusion task force.^126^ Green Thumb Industries Inc stated it would cultivate a diverse culture and workforce through equitable employment, salary, and promotional efforts; we did not find implemented plans.^90^ Innovative Industrial Properties established a diversity, equality, and inclusion policy^124^ but reported no activities.^144^ Trulieve established a diversity, equity, and inclusion task force to build its workforce, partnerships, and events and diversify product suppliers.^97^ Trulieve planned involvement in work fairs and internships to create a more diverse workforce and to train its employees in diversity, equity, and inclusion.^97^ Cresco Labs Inc hosted a business workshop with Michigan’s Marijuana Regulatory Agency as part of the agency’s social equity program. Green Thumb Industries Inc and Cresco Labs Inc used business incubators and licensing programs^91,92,101,102,103,104,113,117,145,146^ to help underrepresented groups establish businesses. Cresco Labs Inc held 13 incubator events in Illinois during 2019 and 2020 that served 255 people and 50 businesses, contributing 2062 staff hours and spending more than $775 000 on licensing fees and incubator events.^119^ Curaleaf Holdings Inc partnered with 1 minority-owned business and Women Grow, an organization supporting women in the cannabis industry, as part of its 420 × 25 supplier diversity initiative seeking to secure 420 product suppliers from underrepresented demographic groups in the cannabis industry by 2025.^75,123,147^ (Curaleaf Holdings Inc claimed 60 community partnerships as of May 2021.^148^) Columbia Care^107^ planned to partner with businesses owned by members of minority groups and women to support social equity initiatives in Illinois^142^ and helped 2 social equity license applicants there develop applications with diversity and community engagement plans after purchasing minority stakes in their enterprises.^140^ Trulieve^132^ and Cresco Labs Inc^132^ sponsored the Historically Black Colleges and Universities Cannabis Equity Initiative seeking to increase Black employment in the cannabis industry. Trulieve provided $15 000 for internships and $20 000 for scholarships administered by the Thurgood Marshall College Fund, the largest Black college organization serving 47 member institutions, to prepare students for cannabis industry careers.^134^ Trulieve and Cresco Labs Inc claimed involvement with National Cannabis Roundtable efforts emphasizing improving industry equity.^96^ Four companies promoted to special populations by partnering with nonprofits, participating in events, and fundraising using special products. Canopy Growth Corporation^125^ and Trulieve^139^ sponsored or participated in LGBTQ celebrations. Trulieve sold special edition products, including limited edition Pride products^136,137^ and TruSwag,^138^ to raise funds for LGBTQ organizations. Curaleaf Holdings Inc contributed to social justice, equity, and women’s organizations,^122^ and Canopy Growth Corporation^128,129^ and Green Thumb Industries Inc^130^ participated in campaigns addressing systemic racism, usually with business coalitions.

**Table 3.** Overview of CSR Activities Between 2012 and 2021 Regarding Diversity, Equity, and Inclusion of the 9 Largest Multinational Cannabis Companies as of January 2021^a^

Company	CSR activity									
	Business incubators or business assistance	Scholarships, internships, and college certification programs	Brands with dedicated DEI funding	Donations from limited-edition product sales or product launches	Participation in DEI events	Internal DEI initiatives and policies	CSR programs with DEI focus	Job training	Joined trade associations or corporate pacts promoting DEI	Supplier diversity and business partnership initiatives
Curaleaf Holdings Inc	Curaleaf Holdings Inc,^121^ 2020	NA	NA	PR Newswire,^122^ 2020	NA	Curaleaf Holdings Inc,^121^ 2021	Curaleaf Holdings Inc,^121^ 2021	NA	NA	Ravilojan,^80^ 2020; MENAFN,^123^ 2021; Curaleaf Holdings Inc,^77^ 2021
Innovative Industrial Properties	NA	NA	NA	NA	NA	Business Wire,^124^ 2021	NA	NA	NA	NA
Canopy Growth Corporation	NA	NA	NA	NA	No author,^125^ 2019	Ravilojan,^80^ 2020; Berke and Lee,^126^ 2021; CNW Group Ltd,^127^ 2021	NA	NA	Business Wire,^128^ 2020; Lee and Dobby,^129^ 2020	NA
Green Thumb Industries Inc	Hasse,^101^ 2020; GlobeNewswire,^104^ 2020	NA	NA	NA	NA	Green Thumb,^90^ 2021	Green Thumb,^90^ 2021	NA	GlobeNewswire,^130^ 2021	NA
Cresco Labs Inc	Business Wire,^91^ 2020; LARA Communications,^131^ 2021	+ Beard,^132^ 2020	NA	NA	No author,^133^ 2020	NA	+No author,^91^ 2020	NA	No author,^96^ 2020	NA
Trulieve	NA	Beard,^132^ 2020; PR Newswire,^134^ 2021; Cision,^135^ 2019	NA	Newstex Blogs,^136^ 2019; Cision PR ewswire,^137^ 2020; PR Newswire,^138^ 2020	GlobeNewswire,^139^ 2020	Trulieve,^97^ 2021	NA	NA	MENAFN,^96^ 2020	Trulieve,^97^ 2021
Cronos Group Inc	NA	NA	NA	NA	NA	NA	NA	NA	NA	NA
GrowGeneration Corp	NA	NA	NA	NA	NA	NA	NA	NA	NA	NA
Columbia Care	No author,^140^ 2020	NA	NA	NA	NA	+ Ponieman,^141^ 2021	NA	Ponieman,^141^ 2021	NA	Business Wire,^142^ 2019

Abbreviations: CSR, corporate social responsibility; DEI, diversity, equity, and inclusion; MENAFN, Middle East and North Africa Financial Network; NA, not applicable.

^a^ Citations link to company involvement in CSR activity.

### Making Charitable Contributions

Seven companies made charitable contributions at national, state, and local levels (eTable 2 in the Supplement).^76,79,100,149,150,151,152,153,154,155,156,157,158,159,160,161,162,163,164,165,166,167,168,169,170,171,172,173,174,175,176,177,178,179,180,181,182,183,184,185,186,187,188,189,190,191^ Canopy Growth Corporation’s^173^ national efforts focused on fundraising for education,^173^ breast cancer,^154,155^ mental health,^192^ and veterans.^79,169^ Curaleaf Holdings Inc targeted breast cancer,^154,155^ selling pink “Pre-rolls With a Purpose” and pink vaporizer pens with plans to dedicate some proceeds to breast cancer nonprofit organizations in 9 states.^155^ Curaleaf Holdings Inc joined the Ice Bucket Challenge to raise funds for research and treatment of amyotrophic lateral sclerosis, sold limited edition Rhythm for a Cause vaporizer pens through its dispensaries, and partnered with organizations in 3 states to promote breast cancer awareness.^182^ Green Thumb Industries Inc indicated it would donate store opening proceeds to the National Giving Alliance, dedicated to helping low-income and homeless persons.^181^ Green Thumb Industries Inc donated more than $34 000 from Dogwalker product sales to 5 animal shelters^183^ and partnered with American Corporate Partners, an organization helping veterans transition to civilian life.^179^ GrowGeneration Corp announced a partnership with Whole Food’s Whole Cities Foundation to donate hydroponics to local community gardens in the US.^191^ As part of Cresco Lab Inc’s Make a Difference initiative, employees participated in 75 activities that the company claimed benefitted more than 50 communities across 8 states.^188,193^ Five companies assisted COVID-19 pandemic relief efforts during 2020. Canopy Growth Corporation gave $20 000 to Wounded Warriors Canada’s trauma therapy programs and its mental health assistance related to COVID-19.^79^ Canopy Growth Corporation also donated personal protective equipment, converted facilities to produce hand sanitizer, and gave Ontario, Canada, 40 000 surgical masks and 25 000 N95 masks. Canopy Growth Corporation’s subsidiary BioSteel Sports Nutrition Inc contributed $2 million in hydration mix to US and Canadian emergency workers.^176,177,178^ Curaleaf Holdings Inc gave frontline workers food.^76^ Trulieve donated more than 150 computers to assist distance learning in racial and ethnic minority communities.^189^ Cresco Labs Inc pledged to hire 250 COVID-19–affected workers, and stated it would pay employees extra during the pandemic.^100^ GrowGeneration Corp pledged as much as $500 000 in equipment to communities and customers affected by COVID-19.^190^ Canopy Growth Corporation, Curaleaf Holdings Inc, Green Thumb Industries Inc, Innovative Industrial Properties, and Trulieve announced or participated in local philanthropic initiatives, including job training,^158,172^ community causes,^135,159,160,161,162,171,187,189^ food banks,^149,150,151,163,164,165,166,167,180,184,194^ homeless shelters,^168^ women’s services,^195^ a campaign to raise funds for Ronald McDonald charities in Canada^170^ (founded by and affiliated with executives of the McDonald’s Corporation, a transnational fast food company^196,197^), animal shelters,^183,184,185^ veteran organizations,^153^ a reforestation organization,^152,198,199^ housing,^200^ and social equity and antipoverty nonprofits.^76,179,188,193,194^ Curaleaf Holdings Inc^76,149^ and Green Thumb Industries Inc^179,180,184,195^ planned or claimed to donate proceeds from retail store openings to local charities. Two companies incorporated their CSR programs, with Curaleaf Holdings Inc announcing it would contribute to Black Owned Maine’s family relief program as part of its Rooted in Good platform and store opening,^76^ and to food charities as part of its Feed the Block initiative.^76,149,150,151^ Trulieve employees locally volunteered for its Make a Difference initiative.^188^

### Researching Potential Therapeutic Uses of Cannabis and Increasing Access to Medical Cannabis

Two companies promoted cannabis’ medical utility (eTable 1 in the Supplement). Canopy Growth Corporation funded research investigating whether medical cannabis treated sleep disorders^201^ or mental health conditions.^192,202^ Canopy Growth Corporation and Columbia Care funded studies on whether cannabis could serve as therapy for opioid misuse^79,202^ or an alternative pain treatment.^203^ Canopy Growth Corporation offered cannabis education programs for physicians from at least 2016 to 2019^204,205,206^ and partnered with the Canadian AIDS Society, an organization representing local Canadian AIDS and HIV groups,^207^ to develop medical cannabis treatment protocols for chronic pain.^79,208^ Canopy Growth Corporation partnered with the Beckley Foundation, a drug reform and psychedelic research think tank,^209^ to form Beckley Canopy Therapeutics to research cannabis-based medicines.^208^ Three companies sought expanded medical access. Trulieve’s TruVet connected veterans with medical cannabis prescribers.^210^ Curaleaf Holdings Inc planned to fund the Veterans Cannabis Project, a nonprofit organization increasing veteran access to medical cannabis,^211,212^ by dedicating proceeds from branded prerolls.^213^ The nonprofit identified Curaleaf Holdings Inc as 1 of 3 partners.^214^ Canopy Growth Corporation funded a national patient survey conducted in July and August 2020 in partnership with Medical Cannabis Canada,^79^ a volunteer-run medical cannabis access nonprofit.^215^ Canopy Growth Corporation sought the survey to lower medical use barriers and gain access to academic institutions, nonprofit organizations, and government officials.^79^

### Efforts That Claimed to Address the Harms of Cannabis Legalization

Three companies used CSR activities that they claimed combatted youth use or drugged driving (Table 4).^77,79,124,216,217,218,219,220,221,222,223,224,225,226,227,228^ Cronos Group Inc^226,227^ and Cresco Labs Inc^222^ developed marketing regulations that they claimed reduced advertising exposure and appeal to youths. Canopy Growth Corporation developed youth prevention materials with Canadian Students for Sensible Drug Policy and Parent Action on Drugs.^79,219,220,221^ Canadian Students for Sensible Drug Policy was a former chapter of international grassroots drug policy reform organization^229^ Students for Sensible Drug Policy.^230,231^ Parent Action on Drugs, which was created in 1983 to prevent use of drugs and alcohol by youths, disbanded in 2019 due to insufficient funding.^174^ Canopy Growth Corporation framed its youth prevention programming as helping youths^221^ and “young adults make healthy, responsible decisions on the use of cannabis.”^220^ Canopy Growth Corporation also partnered with Mothers Against Drunk Driving Canada to sponsor advertisements discouraging cannabis-influenced driving.^79,219,220,221^

**Table 4.** Overview of CSR Activities Between 2012 and 2021 to Address Cannabis Legalization Harms Among the 9 Largest Multinational Cannabis Companies as of January 2021^a^

Company	Sustainability	Youth use prevention	Youth marketing prevention	Drugged driving prevention
Curaleaf Holdings Inc	Curaleaf Holdings Inc,^77^ 2021	NA	NA	NA
Innovative Industrial Properties	Business Wire,^124^ 2021	NA	NA	NA
Canopy Growth Corporation	PR Newswire,^216^ 2019; Agriculture XPRT,^217^ 2019; Canada NewsWire,^218^ 2020	Canopy Growth Corporation,^79^ ND; Rivera,^219^ 2017; no author,^220^ 2018; PR Newswire,^221^ 2019	NA	Canopy Growth Corporation,^79^ ND; Rivera,^219^ 2017; no author,^220^ 2018; PR Newswire,^221^ 2019
Green Thumb Industries Inc	NA	NA	NA	NA
Cresco Labs Inc	NA	NA	Cresco Labs Inc,^222^ 2020	NA
Trulieve	Sustainable Cannabis Coalition,^223^ 2021; PR Newswire, ^224^ 2021; Berke and Lee,^225^ 2021	NA	NA	NA
Cronos Group Inc	Canada NewsWire,^218^ 2020	NA	Cronos Group,^226^ 2021; Cronos Group, ^227^ ND	NA
GrowGeneration Corp	Sustainable Cannabis Coalition,^223^ 2021; PR Newswire, ^224^ 2021; Berke and Lee,^225^ 2021; PR Newswire,^228^ 2021	NA	NA	NA
Columbia Care	NA	NA	NA	NA

Abbreviations: CSR, corporate social responsibility; NA, not applicable; ND, no date.

^a^ Citations link to company involvement in CSR activity.

Six companies claimed they were mitigating their environmental impacts by reducing product waste^175^ and pollution.^232,233^ Curaleaf Holdings Inc formed a sustainability committee.^77^ Trulieve and GrowGeneration Corp were among 17 cofounders of the Sustainable Cannabis Coalition trade association.^223,224,225,228^ In 2019, Canopy Growth Corporation partnered with recycling company TerraCycle to pilot a recycling program for cannabis packaging in Canada, collecting more than 1 million pieces of waste.^216,217^ In 2020, Canopy Growth Corporation and Cronos Group Inc piloted a program with the Cannabis Council of Canada to collect cannabis vaping devices for recycling.^218^ Curaleaf Holdings Inc and Green Thumb Industries Inc listed sustainability as CSR program pillars.^90,121^ Innovative Industrial Properties, which sells real estate to medical cannabis companies,^48,49^ claimed it was sustainable because it reused existing properties over construction.^124^

## Discussion

Our results suggest CSR activities of cannabis companies are similar to those of the tobacco industry, which enabled the latter to recruit customers and allies, encourage consumption, expand markets, legitimize its product, and deter regulation. Cannabis company donations to and partnerships with advocacy organizations could generate goodwill and consumption among minority and LGBTQ+ communities, a tactic tobacco companies have used to market to those groups.^234,235,236^ Collaboration with and funding of advocacy organizations have also been used historically by tobacco companies to form partnerships that allowed them to build policy coalitions supporting their agenda^27,236,237^ (eg, opposing taxes). Cannabis companies also created business partnerships that could expand their reach. Although social equity programs developed by state governments allocate licenses to communities and individuals impacted by criminalization,^238^ they often impose regulatory and financial barriers.^239,240,241^ Proof currently exists that some cannabis companies assisted social equity license applicants in exchange for control of proposed businesses,^238,242,243,244^ providing the businesses with increased market access. Cannabis companies have publicized CSR activities similar to those of the tobacco industry, which has previously used such concerns to generate reasons for policy engagement with government officials.^27,41,245^ Industry research on cannabis as an opioid substitute and to treat mental health disorders and insomnia mirrored tobacco industry pharmaceuticalization,^32,246^ the strategy of selling nicotine replacement therapy to legitimize tobacco company products as therapeutic.^32^ Pharmaceuticalization may normalize and promote cannabis as a health or medical treatment akin to the tobacco industry’s sale of nicotine replacement therapy, possibly providing cannabis companies additional markets to increase consumption and profits. Cannabis company CSR activities regarding youth prevention, cannabis-influenced driving, and sustainability shared commonalities with CSR activities of the tobacco industry, portraying companies as addressing harmful business effects while sidestepping concerns. The tobacco industry has used youth prevention,^28,29^ marketing,^28,247^ and environmental^248^ CSR programs to displace effective educational programs and regulation.^249^ Cannabis companies stated that youth prevention programming helped prevent preteen cannabis use^220,221^ without messaging against consumption and promoted recycling while avoiding occupational risks arising from workplace exposure to toxins^250,251^ and secondhand smoke exposure where cannabis is consumed.^252,253^ Prevention programming directed at youths by the tobacco industry was historically less effective than government prevention programming and promoted youth consumption via forbidden fruit messaging.^28,29^ In the US, youths had a 12.8% rate of current cannabis use compared with 15.4% rate of electronic cigarette use and a 3.3% rate of combustible cigarette use in January through June 2021.^254^ These higher prevalence rates warrant public health messaging that discourages use and avoids normalizing consumption. Cannabis industry promotion of recycling and sustainability programs may divert attention from adoption of regulations preventing harmful environmental pollution. A comparable case is tobacco industry campaigns encouraging individuals to pick up cigarette butts,^255^ the largest source of litter globally,^256^ rather than accepting responsibility for manufacturing products that are not environmentally sustainable and modifying production practices.

### Limitations

This study has some limitations. We included cannabis companies with the largest market capitalizations, which are not necessarily reflective of the entire industry. The publicly available materials included in our analysis are likely incomplete. We excluded video broadcasts and social media posts, potentially affecting our results because social media platforms are used by companies to advertise as well as promote their CSR activities.^257^ Despite these limitations, our findings provide detail on CSR strategies pursued by cannabis companies, including similarities with tobacco companies. Further study is warranted regarding cannabis companies’ use of CSR to influence regulation, improve public image, and secure market access. Little or no research on marketing codes, sustainability, employee protections, and diversity within the industry has been performed, including whether incubator programs provide applicants with full business autonomy. Additional research is also needed to address the reasons cannabis companies choose to focus on some issues and exclude others in their CSR activities (eg, increased focus on sustainability and pollution compared with drugged driving by adolescents). Otañez and Vergara^35^ note that additional research using qualitative interviews as well as quantitative employee and customer surveys are needed to understand certain CSR activities by individual businesses. Ju et al^37^ also state that communications staff at cannabis companies should be interviewed to determine CSR intentions and drivers. Forzely^36^ suggested that CSR should be incorporated as part of the 7C Framework to better analyze cannabis industry marketing methods. Additionally, Paul^38^ posited that community infrastructure theory and media systems dependency models may be used to understand development and consumption of CSR messages. These approaches could be expanded in the future to explore CSR foci and intentions among larger companies in a scope similar to that of our study.

## Conclusions

Cannabis legalization is expanding,^258,259^ making understanding how cannabis companies legitimize themselves critical. Industry motivation to increase consumption makes policies difficult to modify once established.^260^ Public health actors have been wary of industry CSR activities, given research demonstrating such programs are ineffectual by design and advance corporate interests.^261^ Similarly, cannabis companies appear to use CSR activities that normalize and legitimize the industry. US cannabis companies have sought to open markets and influence regulations,^262,263,264^ and Canadian companies have attempted to reduce taxes and regulation,^10,265^ making societal approval critical. Public health proponents should challenge these attempts to influence policy and adopt strong protective measures.

## Notes

Supplement. **eTable 1.** Overview of CSR Activities Between 2012 and 2021 Regarding Medical Cannabis Access and Utility as a Treatment of the 9 Largest Multinational Cannabis Companies as of January 2021 **eTable 2.** Overview of General CSR Activity Categories and Charity Focuses Between 2012 and 2021 Among the 9 Largest Multinational Cannabis Companies as of January 2021

## References

[1] NORML. Legalization. 2021. Accessed October 18, 2021. https://norml.org/laws/legalization/

[2] United States Drug Enforcement Administration. Drug Scheduling. Accessed October 18, 2021. https://www.dea.gov/drug-information/drug-scheduling

[3] Borgelt LM, Franson KL, Nussbaum AM, Wang GS. The pharmacologic and clinical effects of medical cannabis. Pharmacotherapy. 2013;33(2):195-209. doi:10.1002/phar.1187 23386598

[4] Nugent SM, Morasco BJ, O’Neil ME, . The effects of cannabis among adults with chronic pain and an overview of general harms: a systematic review. Ann Intern Med. 2017;167(5):319-331. doi:10.7326/M17-0155 28806817

[5] Subritzky T, Lenton S, Pettigrew S. Legal cannabis industry adopting strategies of the tobacco industry. Drug Alcohol Rev. 2016;35(5):511-513. doi:10.1111/dar.12459 27650812

[6] Barry RA, Glantz SA. Marijuana regulatory frameworks in four US states: an analysis against a public health standard. Am J Public Health. 2018;108(7):914-923. doi:10.2105/AJPH.2018.304401 29874509 PMC5993386

[7] Caulkins JP, Kilborn ML. Cannabis legalization, regulation, & control: a review of key challenges for local, state, and provincial officials. Am J Drug Alcohol Abuse. 2019;45(6):689-697. doi:10.1080/00952990.2019.1611840 31135218

[8] Government of Canada. What you need to know about cannabis. Updated July 29, 2021. Accessed November 18, 2021. https://web.archive.org/web/20211104174655/https://www.canada.ca/en/services/health/campaigns/cannabis/canadians.html

[9] Government of Canada. A framework for the legalization and regulation of cannabis in Canada. November 30, 2016. Accessed November 18, 2021. https://www.canada.ca/en/health-canada/services/drugs-medication/cannabis/laws-regulations/task-force-cannabis-legalization-regulation/framework-legalization-regulation-cannabis-in-canada.html

[10] CBC. The pros, cons and unknowns of legal cannabis in Canada 3 years later. Updated October 26, 2021. Accessed November 18, 2021. https://www.cbc.ca/news/politics/cannabis-changed-canada-1.6219493

[11] Cannabis Legalization in the US. Population Health Impacts. Project HOPE; 2021.

[12] Martins SS, Segura LE, Levy NS, . Racial and ethnic differences in cannabis use following legalization in US states with medical cannabis laws. JAMA Netw Open. 2021;4(9):e2127002. doi:10.1001/jamanetworkopen.2021.27002 34570205 PMC8477268

[13] Fischer B, Lee A, Robinson T, Hall W. An overview of select cannabis use and supply indicators pre- and post-legalization in Canada. Subst Abuse Treat Prev Policy. 2021;16(1):77. doi:10.1186/s13011-021-00405-7 34620191 PMC8496143

[14] National Institute on Drug Abuse. What is the scope of cannabis (marijuana) use in the United States? July 2020. Accessed June 2, 2022. https://nida.nih.gov/publications/research-reports/marijuana/what-scope-marijuana-use-in-united-states

[15] Government of Canada. Canadian Cannabis Survey 2020: summary. Updated August 12, 2021. Accessed November 17, 2021. https://www.canada.ca/en/health-canada/services/drugs-medication/cannabis/research-data/canadian-cannabis-survey-2020-summary.html

[16] Dorfman L, Cheyne A, Friedman LC, Wadud A, Gottlieb M. Soda and tobacco industry corporate social responsibility campaigns: how do they compare? PLoS Med. 2012;9(6):e1001241. doi:10.1371/journal.pmed.1001241 22723745 PMC3378589

[17] Herrick C. Shifting blame/selling health: corporate social responsibility in the age of obesity. Sociol Health Illn. 2009;31(1):51-65. doi:10.1111/j.1467-9566.2008.01121.x 18764803

[18] Gehman J, Lefsrud LM, Fast S. Social license to operate: legitimacy by another name? Can Public Adm. 2017;60(2):293-317. doi:10.1111/capa.12218

[19] Shiu YM, Yang SL. Does engagement in corporate social responsibility provide strategic insurance-like effects? Strateg Manage J. 2017;38(2):455-470. doi:10.1002/smj.2494

[20] Jeffrey S, Rosenberg S, McCabe B. Corporate social responsibility behaviors and corporate reputation. Soc Responsib J. 2018;15(3):395-408. doi:10.1108/SRJ-11-2017-0255

[21] Palazzo G, Richter U. CSR business as usual? the case of the tobacco industry. J Bus Ethics. 2005;61(4):387-401. doi:10.1007/s10551-005-7444-3

[22] Grougiou V, Dedoulis E, Leventis S. Corporate social responsibility reporting and organizational stigma: the case of “sin” industries. J Bus Res. 2016;69(2):905-914. doi:10.1016/j.jbusres.2015.06.041

[23] Hudson BA. Against all odds: a consideration of core-stigmatized organizations. Acad Manage Rev. 2008;33(1):252-266. doi:10.5465/amr.2008.27752775

[24] Hirschhorn N. Corporate social responsibility and the tobacco industry: hope or hype? Tob Control. 2004;13(4):447-453. doi:10.1136/tc.2003.006676 15564636 PMC1747956

[25] World Health Organization. Tobacco industry and corporate responsibility ... an inherent contradiction. 2017. Accessed September 8, 2021. https://escholarship.org/uc/item/6kf7q7v9#main

[26] Yoon S, Lam TH. The illusion of righteousness: corporate social responsibility practices of the alcohol industry. BMC Public Health. 2013;13(1):630. doi:10.1186/1471-2458-13-630 23822724 PMC3706248

[27] Fooks GJ, Gilmore AB. Corporate philanthropy, political influence, and health policy. PLoS One. 2013;8(11):e80864. doi:10.1371/journal.pone.0080864 24312249 PMC3842338

[28] Landman A, Ling PM, Glantz SA. Tobacco industry youth smoking prevention programs: protecting the industry and hurting tobacco control. Am J Public Health. 2002;92(6):917-930. doi:10.2105/AJPH.92.6.917 12036777 PMC1447482

[29] Apollonio DE, Malone RE. The “We Card” program: tobacco industry “youth smoking prevention” as industry self-preservation. Am J Public Health. 2010;100(7):1188-1201. doi:10.2105/AJPH.2009.169573 20466965 PMC2882417

[30] Smith EA, Malone RE. “We will speak as the smoker”: the tobacco industry’s smokers’ rights groups. Eur J Public Health. 2007;17(3):306-313. doi:10.1093/eurpub/ckl244 17065174 PMC2794244

[31] American Nonsmokers’ Rights Foundation. National Smokers Alliance exposed. Updated January 1999. Accessed November 3, 2021. https://no-smoke.org/wp-content/uploads/pdf/nsa_exposed.pdf

[32] Hendlin YH, Elias J, Ling PM. The pharmaceuticalization of the tobacco industry. Ann Intern Med. 2017;167(4):278-280. doi:10.7326/M17-0759 28715843 PMC5568794

[33] World Health Organization. WHO Framework Convention on Tobacco Control. Updated 2005. Accessed November 2, 2021. http://apps.who.int/iris/bitstream/handle/10665/42811/9241591013.pdf;jsessionid=AFA4F21357E845E9C3107A37521FE08D?sequence=1

[34] World Health Organization. Guidelines for implementation of Article 13: WHO Framework Convention on Tobacco Control (Tobacco advertising, promotion and sponsorship). January 1, 2013. Accessed September 8, 2021. https://fctc.who.int/publications/m/item/tobacco-advertising-promotion-and-sponsorship

[35] Otanez M, Vergara D. Cannabis corporate social responsibility: a critical and mixed method approach. In: Corva D, Meisel JS, eds. The Routledge Handbook of Post-Prohibition Cannabis Research. Routledge; 2021:9. Accessed May 27, 2022. https://www.taylorfrancis.com/chapters/edit/10.4324/9780429320491-19/cannabis-corporate-social-responsibility-marty-ota%C3%B1ez-david-vergara?context=ubx&refId=b7ea88af-4dfe-4a8b-9beb-8d07e1996c37

[36] Forzley S. Cannabis and web marketing strategies—a study of Colorado cannabis vendors. Br Food J. 2021;123(11):3393-3403. doi:10.1108/BFJ-04-2019-0247

[37] Ju R, Dong C, Zhang Y. How controversial businesses communicate CSR on Facebook: insights from the Canadian cannabis industry. Public Relat Rev. 2021;47(4):102059. doi:10.1016/j.pubrev.2021.102059

[38] Paul K. Stakeholder theory, meet communications theory: media systems dependency and community infrastructure theory, with an application to California’s cannabis/marijuana industry. J Bus Ethics. 2015;129(3):705-720. doi:10.1007/s10551-014-2168-x

[39] CBC. A legal history of smoking in Canada. CBC/Radio-Canada. Updated November 19, 2012. Accessed June 3, 2022. https://www.cbc.ca/news/health/a-legal-history-of-smoking-in-canada-1.982213

[40] Gershon L. A brief history of tobacco in America. *JSTOR Daily*. June 10, 2016. Accessed June 3, 2022. https://daily.jstor.org/a-brief-history-of-tobacco-in-america/

[41] Fooks GJ, Gilmore AB, Smith KE, Collin J, Holden C, Lee K. Corporate social responsibility and access to policyélites: an analysis of tobacco industry documents. PLoS Med. 2011;8(8):e1001076. doi:10.1371/journal.pmed.1001076 21886485 PMC3160341

[42] Williams S. The 10 largest marijuana stocks in 2021. Nasdaq. January 10, 2021. Accessed October 8, 2021. https://www.nasdaq.com/articles/the-10-largest-marijuana-stocks-in-2021-2021-01-10

[43] Hayes A. Investopedia: Nasdaq. Updated May 5, 2022. Accessed October 8, 2021. https://www.investopedia.com/terms/n/nasdaq.asp

[44] Nasdaq Canada. 2021. Accessed December 9, 2021. https://www.nasdaq.com/nasdaqcanada

[45] Curaleaf Holdings Inc. Stock price & news—Google Finance. November 15, 2021. Accessed November 15, 2021. https://www.google.com/finance/quote/CURA:CNSX

[46] Curaleaf Holdings Inc. Investor relations. November 8, 2021. Accessed November 8, 2021. https://ir.curaleaf.com/overview

[47] Innovative Industrial Properties Inc. Stock price & news—Google Finance. November 15, 2021. Accessed November 15, 2021. https://www.google.com/finance/quote/IIPR:NYSE

[48] Innovative Industrial Properties. Our portfolio. Accessed November 5, 2021. https://innovativeindustrialproperties.com/iip-our-portfolio/

[49] Innovative Industrial Properties. Real estate cannabis investment firms. Accessed November 5, 2021. https://innovativeindustrialproperties.com/

[50] Canopy Growth Corp (CGC). Stock price & news—Google Finance. November 15, 2021. Accessed November 15, 2021. https://www.google.com/finance/quote/CGC:NASDAQ

[51] Canopy Growth Corporation: stock performance & earnings. PitchBook. 2021. Accessed November 8, 2021. https://pitchbook.com/profiles/company/59682-52

[52] Green Thumb Industries Inc. Stock price & news—Google Finance. November 15, 2021. Accessed November 15, 2021. https://www.google.com/finance/quote/GTII:CNSX

[53] Green Thumb Industries Inc. Investor relations. 2021. Accessed November 8, 2021. https://investors.gtigrows.com/investors/overview/default.aspx

[54] Cresco Labs Inc. Stock price & news—Google Finance. November 15, 2021. Accessed November 15, 2021. https://www.google.com/finance/quote/CRLBF:OTCMKTS

[55] Investor overview. Cresco Labs. Accessed November 8, 2021. https://investors.crescolabs.com/investors/overview/default.aspx

[56] Trulieve Cannabis Corp. Google Finance. November 15, 2021. Accessed November 15, 2021. https://www.google.com/finance/quote/TCNNF:OTCMKTS

[57] Trulieve. Investor relations. 2018-2021. Accessed November 8, 2021. https://investors.trulieve.com/investors/

[58] Cronos Group Inc. Stock price & news—Google Finance. November 15, 2021. Accessed November 15, 2021. https://www.google.com/finance/quote/CRON:NASDAQ

[59] Cronos Group Inc. Company Profile—CNNMoney. com. 2021. Accessed November 8, 2021. https://money.cnn.com/quote/profile/profile.html?symb=CRON

[60] GrowGeneration Corporation. Frequently asked questions. 2021. Accessed November 8, 2021. https://ir.growgeneration.com/company-information/faq

[61] Columbia Care Inc. Stock price & news—Google Finance. November 15, 2021. Accessed November 15, 2021. https://www.google.com/finance/quote/CCHWF:OTCMKTS

[62] Columbia Care Inc. Frequently asked questions. 2021. Accessed November 8, 2021. https://ir.col-care.com/resources/investor-faqs

[63] Bero L. Implications of the tobacco industry documents for public health and policy. Annu Rev Public Health. 2003;24(1):267-288. doi:10.1146/annurev.publhealth.24.100901.140813 12415145

[64] Malone RE, Balbach ED. Tobacco industry documents: treasure trove or quagmire? Tob Control. 2000;9(3):334-338. doi:10.1136/tc.9.3.334 10982579 PMC1748361

[65] Anderson SJ, McCandless PM, Klausner K, Taketa R, Yerger VB. Tobacco documents research methodology. Tob Control. 2011;20(suppl 2):ii8-ii11. doi:10.1136/tc.2010.041921 21504933 PMC3085001

[66] McDaniel PA, Intinarelli G, Malone RE. Tobacco industry issues management organizations: creating a global corporate network to undermine public health. Global Health. 2008;4(1):2. doi:10.1186/1744-8603-4-2 18201375 PMC2265275

[67] Kostygina G, Ling PM. Tobacco industry use of flavourings to promote smokeless tobacco products. Tob Control. 2016;25(suppl 2):ii40-ii49. doi:10.1136/tobaccocontrol-2016-053212 27856998 PMC5433525

[68] Ross JS, Hill KP, Egilman DS, Krumholz HM. Guest authorship and ghostwriting in publications related to rofecoxib: a case study of industry documents from rofecoxib litigation. JAMA. 2008;299(15):1800-1812. doi:10.1001/jama.299.15.1800 18413874

[69] Neale J. Iterative categorization (IC): a systematic technique for analysing qualitative data. Addiction. 2016;111(6):1096-1106. doi:10.1111/add.13314 26806155 PMC5069594

[70] Emler R. Cannabis company Canopy Growth signs deal with alcohol wholesaler. Drinks Business. April 26, 2021. Accessed October 8, 2021. https://www.thedrinksbusiness.com/2021/04/cannabis-company-canopy-growth-signs-deal-with-alcohol-wholesaler/

[71] Gelles D. When the Makers of Marlboro and Corona get into marijuana. *New York Times*. December 12, 2018. Accessed October 8, 2021. https://www.nytimes.com/2018/12/12/business/cannabis-business-altria-canopy-constellation-cronos.html

[72] Cronos Group. Altria. Accessed October 8, 2021. https://www.altria.com/Overlays/Companies-and-Brands/CRONOS

[73] Curaleaf. B Aware B Invested B Noble. 2021. Accessed September 29, 2021. https://curaleaf.com/bnoble

[74] Curaleaf. Bernard Noble and Fab 5 Freddy partner with Curaleaf to launch B Noble brand. July 13, 2021. Accessed September 1, 2021. https://ir.curaleaf.com/2021-07-13-Bernard-Noble-and-Fab-5-Freddy-Partner-with-Curaleaf-to-Launch-B-Noble-Brand

[75] Benzinga. Curaleaf unveils new hiring initiative focused on social equity on heels of opening 100th store. February 10, 2021. Accessed August 29, 2021. https://www.benzinga.com/markets/cannabis/21/02/19586260/curaleaf-unveils-new-hiring-initiative-focused-on-social-equity-on-heels-of-opening-100th-store

[76] PR Newswire. First Curaleaf-branded adult-use store opens in Maine. April 30, 2021. Accessed September 1, 2021. https://www.prnewswire.com/news-releases/first-curaleaf-branded-adult-use-store-opens-in-maine-301280980.html

[77] Curaleaf. Environmental sustainability. 2021. Accessed September 30, 2021. https://curaleaf.com/sustainability

[78] Middle East and North Africa Financial Network. Canopy, Curaleaf, advocacy groups join forces to advance US cannabis reform. February 9, 2021. Accessed September 5, 2021. https://menafn.com/1101574723/Canopy-Curaleaf-advocacy-groups-join-forces-to-advance-US-cannabis-reform

[79] Canopy Growth Corporation. Corporate social purpose. Accessed September 28, 2021. https://www.canopygrowth.com/about/corporate-social-purpose/

[80] Ravilojan U. New study finds less than 1 per cent of Canadian corporate leaders are Black. July 4, 2020. Accessed September 4, 2021. https://www.thestar.com/business/2020/07/04/less-than-one-per-cent-of-corporate-leaders-at-tsx-60-companies-are-black-researchers-find.html

[81] GlobeNewswire. Green Thumb Industries announces partnership with Last Prisoner Project; releases short documentary, “Waiting to Breathe,” to raise awareness of LPP’s clemency and reentry initiatives. September 1, 2020. Accessed September 1, 2021. https://www.globenewswire.com/news-release/2020/09/01/2086756/0/en/Green-Thumb-Industries-Announces-Partnership-with-Last-Prisoner-Project-Releases-Short-Documentary-Waiting-to-Breathe-to-Raise-Awareness-of-LPP-s-Clemency-and-Reentry-Initiatives.html

[82] Middle East and North Africa Financial Network. Green Thumb Industries calls for nonprofit grant applications as part of Good Green Brand launch. July 21, 2021. Accessed September 3, 2021. https://menafn.com/1102487330/Green-Thumb-Industries-Calls-for-Nonprofit-Grant-Applications-as-Part-of-Good-Green-Brand-Launch

[83] Green Thumb to launch Good Green line to help marginalized communities. theflyonthewall.com. July 21, 2021. Accessed September 1, 2021. https://www.lexisnexis.com/en-us/professional/academic/nexis-uni.page

[84] GlobeNewswire. Green Thumb Industries continues community investment initiatives with scholarships and expanded partnerships to promote diversity in the cannabis industry. February 25, 2021. Accessed September 4, 2021. https://www.globenewswire.com/news-release/2021/02/25/2182272/0/en/Green-Thumb-Industries-Continues-Community-Investment-Initiatives-with-Scholarships-and-Expanded-Partnerships-to-Promote-Diversity-in-the-Cannabis-Industry.html

[85] Green Thumb Industries. Cannabis for all. 2021. Accessed October 7, 2021. https://www.gtigrows.com/people/cannabis-for-all

[86] PR Newswire. An expanding legal marketplace fosters innovation in the cannabis industry. January 20, 2021. Accessed September 3, 2021. https://www.prnewswire.com/news-releases/an-expanding-legal-marketplace-fosters-innovation-in-the-cannabis-industry-301211470.html

[87] Green Thumb Industries trading down 1%, despite announcement to open fifth essence store in Nevada. MT Newswires Live Briefs INVESTOR Canada. June 26, 2020. Accessed September 5, 2021. https://www.lexisnexis.com/en-us/professional/academic/nexis-uni.page

[88] GlobeNewswire. Green Thumb Industries to Open Essence South Durango, its fifth cannabis store in Las Vegas area and 48th in the nation, on June 27. June 26, 2020. Accessed September 5, 2021. https://www.globenewswire.com/news-release/2020/06/26/2053957/0/en/Green-Thumb-Industries-to-Open-Essence-South-Durango-Its-Fifth-Cannabis-Store-in-Las-Vegas-Area-and-48th-in-the-Nation-on-June-27.html

[89] Green Thumb Industries Inc to open Rise Duncansville. June 26, 2020. Accessed September 4, 2021. https://www.lexisnexis.com/en-us/professional/academic/nexis-uni.page

[90] Green Thumb. Growing for good. 2021. Accessed September 29, 2021. https://www.gtigrows.com/people/social-impact

[91] Business Wire. Cresco Labs publishes inaugural seed annual report. December 29, 2020. Accessed September 2, 2021. https://www.businesswire.com/news/home/20201229005072/en/

[92] Cresco Labs. Social Equity and Education Development. 2020. Accessed September 28, 2021. https://www.crescolabs.com/seed/

[93] Business Wire. Cresco Labs launches social justice campaign on 50th anniversary of War on Drugs. June 17, 2021. Accessed September 3, 2021. https://www.businesswire.com/news/home/20210617005199/en/

[94] Business Wire. Cresco Labs announces the opening of its first five Sunnyside* dispensaries and the launch of Sunnyside.shop in preparation for first day of adult-use cannabis sales in Illinois: Sunnyside* stores in Lakeview, Elmwood Park, Champaign and Rockford expect to serve thousands of customers on January 1. December 30, 2019. Accessed September 4, 2021. https://www.businesswire.com/news/home/20191230005090/en/

[95] Business Wire. Cresco Labs launches cannabis industry’s first comprehensive national social equity & education initiative. May 29, 2019. Accessed September 3, 2021. https://www.businesswire.com/news/home/20190529005246/en/

[96] Middle East and North Africa Financial Network. Statement from the National Cannabis Roundtable. June 3, 2020. Accessed September 1, 2021. https://menafn.com/1100267673/Statement-from-the-National-Cannabis-Roundtable

[97] Trulieve. Diversity, equity & inclusion. 2018-2021. Accessed September 28, 2021. https://investors.trulieve.com/corporate-governance/diversity-equity-inclusion

[98] Business Wire. Top cannabis businesses, associations, and advocacy organizations join forces to launch US Cannabis Council: a coalition of leading companies and advocates, USCC aims to advance social equity and racial justice, and end federal cannabis prohibition. February 8, 2021. Accessed September 29, 2021. https://www.businesswire.com/news/home/20210208005220/en/

[99] American Bar Association. What is “expungement?” November 20, 2018. Accessed October 15, 2021. https://www.americanbar.org/groups/public_education/publications/teaching-legal-docs/what-is-_expungement-/

[100] Businesswire. Cresco Labs expanding local workforce to hire displaced workers: providing extra support to employees in response to COVID-19. April 13, 2020. Accessed September 2, 2021. https://www.businesswire.com/news/home/20200413005161/en/

[101] Hasse J. The week in cannabis: moves from the DEA, FDA, USDA; New York Fashion Week; and quarterly reports. yahoo.com. September 1, 2019. Accessed August 30, 2021. https://www.yahoo.com/video/week-cannabis-moves-dea-fda-215632643.html

[102] Green Thumb Industries. License Education Assistance Program. 2021. Accessed September 28, 2021. https://www.gtigrows.com/people/leap-2

[103] GlobeNewswire. Green Thumb Industries opens LEAP new business accelerator applications for social equity licensees in Illinois. July 29, 2021. Accessed August 27, 2021. https://www.globenewswire.com/news-release/2021/07/29/2271472/0/en/Green-Thumb-Industries-Opens-LEAP-New-Business-Accelerator-Applications-for-Social-Equity-Licensees-in-Illinois.html

[104] GlobeNewswire. Green Thumb Industries (GTI) continues its social equity License Education Assistance Program (LEAP) with pro bono mentoring for craft grower, infuser and transporter licenses in Illinois. January 15, 2020. Accessed September 1, 2021. https://www.globenewswire.com/news-release/2020/01/15/1970785/0/en/Green-Thumb-Industries-GTI-Continues-its-Social-Equity-License-Education-Assistance-Program-LEAP-with-Pro-Bono-Mentoring-for-Craft-Grower-Infuser-and-Transporter-Licenses-in-Illino.html

[105] GlobeNewswire. Green Thumb Industries launches Illinois pro bono cannabis license application assistance program to assist social equity applicants. August 27, 2019. Accessed September 2, 2021. https://www.globenewswire.com/en/news-release/2019/08/27/1906975/0/en/Green-Thumb-Industries-Launches-Illinois-Pro-Bono-Cannabis-License-Application-Assistance-Program-to-Assist-Social-Equity-Applicants.html

[106] Albarazi H. Greenspoon Marder nabs Ex-Locke Lord cannabis law attys. July 7, 2021. Accessed September 2, 2021. https://www.law360.com/articles/1400437/greenspoon-marder-nabs-ex-locke-lord-cannabis-law-attys

[107] Business Wire. Cresco Labs launches first industry-defining incubator program for Illinois adult-use market dispensary applicants: program provides actionable, expert guidance to bring inclusivity to cannabis industry. November 6, 2019. Accessed September 2, 2021. https://www.businesswire.com/news/home/20191106006081/en/

[108] Good Green. Good Green in the community. 2021. Accessed November 18, 2021. https://good.green/grant-recipients#top

[109] Steve DeAngelo. 2020. Accessed November 3, 2021. https://stevedeangelo.com/

[110] LinkedIn. Andrew DeAngelo. 2020. Accessed November 3, 2021. https://www.linkedin.com/in/andrewdeangelo/

[111] Last Prisoner Project. Our programs. 2021. Accessed November 3, 2021. https://web.archive.org/web/20210326151113/https://www.lastprisonerproject.org/programs

[112] Last Prisoner Project. Frequently asked questions. 2021. Accessed November 3, 2021. https://www.lastprisonerproject.org/faqs

[113] Singh A, Chang J. Turning point: cannabis and justice for all. ABC News Nightline. June 1, 2021. Accessed July 22, 2022. https://abc.com/shows/nightline/episode-guide/2021-06/01-tuesday-june-1-2021

[114] Cresco Labs establishes its social equity & educational development program. University of California San Francisco. June 5, 2019. Accessed September 1, 2021. https://www.lexisnexis.com/en-us/professional/academic/nexis-uni.page

[115] Middle East and North Africa Financial Network. The Cleveland School of Cannabis announces scholarship to promote equity in the cannabis industry. March 16, 2021. Accessed August 29, 2021. https://menafn.com/1101761071/The-Cleveland-School-of-Cannabis-Announces-Scholarship-to-Promote-Equity-in-the-Cannabis-Industry

[116] Royse D. Cresco Labs CEO: the future of cannabis legalization includes social equity. yahoo.com. October 22, 2019. Accessed September 1, 2021. https://www.yahoo.com/lifestyle/cresco-labs-ceo-future-cannabis-190315224.html

[117] Cresco Labs highlights Illinois expansion plan to serve adult use cannabis market. University of California San Francisco. June 28, 2019. Accessed September 4, 2021. https://www.lexisnexis.com/en-us/professional/academic/nexis-uni.page

[118] Hasse J. Akerna CEO Jessica Billingsley’s newest challenge: leading the US Cannabis Council. August 11, 2021. Accessed September 2, 2021. https://www.benzinga.com/markets/cannabis/21/08/22447935/akerna-ceo-jessica-billingsleys-newest-challenge-leading-the-us-cannabis-council

[119] Cresco Labs. SEED 2019/2020 annual report. Accessed November 3, 2021. https://www.crescolabs.com/wp-content/uploads/2021/01/122820_SEED_AnnualReport_WebVersion_LR.pdf

[120] GlobeNewswire. Green Thumb Industries to open Rise Kendall, its 50th retail location, on November 18. November 11, 2020. Accessed September 5, 2021. https://www.globenewswire.com/news-release/2020/11/11/2124616/0/en/Green-Thumb-Industries-to-Open-Rise-Kendall-Its-50th-Retail-Location-on-November-18.html

[121] Curaleaf. Social responsibility. 2021. Accessed September 29, 2021. https://curaleaf.com/social-responsibility/

[122] PR Newswire. Curaleaf’s Select Brand expands into New York. September 22, 2020. Accessed September 1, 2021. https://www.prnewswire.com/news-releases/curaleafs-select-brand-expands-into-new-york-301135626.html

[123] Middle East and North Africa Financial Network. “Fourtwenty Collections’’ OMG deity bars now available at Curaleaf. May 28, 2021. Accessed September 1, 2021. https://menafn.com/1102165189/Fourtwenty-Collections-OMG-Deity-Bars-Now-Available-at-Curaleaf

[124] Business Wire. Innovative industrial properties publishes inaugural environmental, social and corporate governance (ESG) report. June 8, 2021. Accessed August 28, 2021. https://www.businesswire.com/news/home/20210608005206/en/

[125] Canopy Growth - Tweed joins Pride Toronto to support progress, champion diversity and celebrate love. ENP Newswire. June 24, 2019. Accessed August 24, 2021. https://www.lexisnexis.com/en-us/professional/academic/nexis-uni.page

[126] Berke J, Lee YJ. Top executives at the 14 largest cannabis companies are overwhelmingly white men, an Insider analysis shows. June 30, 2021. Accessed September 29, 2021. https://www.businessinsider.com/cannabis-industry-diversity-executives-are-white-male-insider-inequity-analysis-shows-2021-6#:~:text=White%20men%20make%20up%2070,across%20a%20range%20of%20sectors

[127] CNW Group Ltd. Sumayyah Emeh-edu joins Canopy Growth as Vice President of Diversity, Equity, and Inclusion. March 31, 2021. Accessed September 2, 2021. https://www.newswire.ca/news-releases/sumayyah-emeh-edu-joins-canopy-growth-as-vice-president-of-diversity-equity-and-inclusion-843400054.html

[128] Business Wire. Canadian CEOs Commemorate Launch of the BlackNorth Initiative. July 20, 2020. Accessed August 20, 2021. https://www.businesswire.com/news/home/20200720005830/en/

[129] Lee YJ, Dobby C. Pledge to end systemic racism at Canadian companies picks up steam. July 20, 2020. Accessed September 2, 2021. https://www.theglobeandmail.com/business/article-pledge-to-end-systemic-racism-at-canadian-companies-picks-up-steam/

[130] GlobeNewswire. Stephen Curry and leading organizations across US join NinetyToZero to combat racial wealth gap. July 15, 2021. Accessed September 5, 2021. https://www.globenewswire.com/en/news-release/2021/07/15/2263876/0/en/Stephen-Curry-and-Leading-Organizations-Across-U-S-Join-NinetyToZero-to-Combat-Racial-Wealth-Gap.html

[131] LARA Communications. MRA’s social equity program announces February Business Resource Workshop, featuring Cresco Labs. Cannabis Regulatory Agency. January 29, 2021. Accessed August 29, 2021. https://www.michigan.gov/cra/spotlight/mras-social-equity-program-announces-february-business-resource-workshop-featuring-cresco-labs

[132] Beard TA. FAMU, other HBCUs seek diversity in cannabis industry. September 2, 2020. Accessed September 1, 2021. http://www.thefamuanonline.com/2020/09/02/famu-other-hbcus-seek-diversity-in-cannabis-industry/

[133] Business Wire. Cresco Labs recognized by Clio Cannabis for marketing creative excellence. December 22, 2020. Accessed September 5, 2021. https://www.businesswire.com/news/home/20201222005193/en/

[134] PR Newswire. Trulieve partners with the Thurgood Marshall College Fund to provide college scholarships. February 26, 2021. Accessed August 20, 2021. https://www.prnewswire.com/news-releases/trulieve-partners-with-the-thurgood-marshall-college-fund-to-provide-college-scholarships-301236619.html

[135] Cision. Trulieve to receive Diversity and Inclusion Champion of the Year award. January 29, 2019. Accessed August 22, 2021. https://www.newswire.ca/news-releases/trulieve-to-receive-diversity-and-inclusion-champion-of-the-year-award-862292316.html

[136] Newstex Blogs. Trulieve, Equality Florida partner for Pride Month. June 17, 2019. Accessed August 29, 2021. https://floridapolitics.com/archives/298999-pride-month-partnership-trulieve/

[137] Cision PR Newswire. Trulieve launches Edition Cartridge, partners with Florida-based LGBTQ+ organizations for Pride Month. June 1, 2020. Accessed September 2, 2021. https://www.prnewswire.com/news-releases/trulieve-launches-limited-edition-cartridge-partners-with-florida-based-lgbtq-organizations-for-pride-month-301068790.html

[138] PR Newswire. Trulieve celebrates Florida’s LGBTQ+ community with $50K donation. July 8, 2020. Accessed September 1, 2021. https://www.prnewswire.com/news-releases/trulieve-celebrates-floridas-lgbtq-community-with-50k-donation-301089980.html

[139] GlobeNewswire. KindRED Pride Foundation announces global campaign to get one million people to wear red on the first Saturday in June to celebrate the 30th anniversary of Gay Disney. February 20, 2020. Accessed September 5, 2021. https://www.globenewswire.com/news-release/2020/02/20/1988090/0/en/KindRED-Pride-Foundation-Announces-Global-Campaign-to-Get-One-Million-People-to-Wear-Red-on-the-First-Saturday-in-June-to-Celebrate-the-30th-Anniversary-of-Gay-Disney.html#:~:text=20%2C%202020%20(GLOBE%20NEWSWIRE),diversity%2C%20inclusion%2C%20equality%2C%20safe

[140] Columbia Care updates on growth: health and beauty close-up. January 9, 2020. Accessed August 29, 2021. https://www.lexisnexis.com/en-us/professional/academic/nexis-uni.page

[141] Ponieman N. Columbia Care CEO, Ayr Wellness COO talk up NJ’s new recreational cannabis market. yahoo.com. February 25, 2021. Accessed August 29, 2021. https://www.yahoo.com/now/columbia-care-ceo-ayr-wellness-214410386.html

[142] Business Wire. Columbia Care completes consolidation of minority interest in Illinois operations. August 15, 2019. Accessed August 27, 2021. https://www.businesswire.com/news/home/20190815005655/en/

[143] Curaleaf. Diversity, equity & inclusion. 2021. Accessed September 29, 2021. https://curaleaf.com/diversity-equity-inclusion

[144] Innovative Industrial Properties Inc. Diversity, equality and inclusion policy statement. Accessed November 4, 2021. http://investors.innovativeindustrialproperties.com/~/media/Files/I/IIP-IR/documents/governance-documents/diversity-equality-and-inclusion-policy-statement-4-14-21.pdf

[145] SocialBizWire. Cresco Labs program provides actionable, expert guidance to bring inclusivity to Cannabis Industry. November 7, 2019. Accessed August 28, 2021. https://www.lexisnexis.com/en-us/professional/academic/nexis-uni.page

[146] Zdinjak N. Cresco Labs kicks off its community impact incubator program. November 7, 2019. Accessed August 27, 2021. https://www.yahoo.com/now/cresco-labs-kicks-off-community-145817098.html

[147] Curaleaf. Women Grow and Curaleaf partner to further “rooted in good” supplier diversity initiatives. 2021. Accessed November 10, 2021. https://curaleaf.com/blog/

[148] Q1 2021 Curaleaf Holdings Inc Earnings Call—Final. Accessed November 10, 2021. https://www.lexisnexis.com/en-us/professional/academic/nexis-uni.page

[149] PR Newswire. Curaleaf opens two new retail locations in Greater Philadelphia and finalizes rebrand of nine retail locations in Pennsylvania. April 8, 2021. Accessed August 27, 2021. https://www.prnewswire.com/news-releases/curaleaf-opens-two-new-retail-locations-in-greater-philadelphia-and-finalizes-rebrand-of-nine-retail-locations-in-pennsylvania-301264804.html

[150] Canada NewsWire. Curaleaf announces “Feed the Block” program to address hunger in several states. November 23, 2020. Accessed August 30, 2021. https://www.newswire.ca/news-releases/curaleaf-announces-feed-the-block-program-to-address-hunger-in-several-states-849790144.html

[151] Canada NewsWire. First Curaleaf-branded retail opens in Harrisburg, Pennsylvania. January 29, 2021. Accessed August 28, 2021. https://www.newswire.ca/news-releases/first-curaleaf-branded-retail-location-opens-in-harrisburg-pennsylvania-839511899.html

[152] Curaleaf. Curaleaf expands presence in New Jersey with new dispensary and cultivation facility. June 24, 2021. Accessed August 30, 2021. https://ir.curaleaf.com/2021-06-24-Curaleaf-Expands-Presence-in-New-Jersey-with-New-Dispensary-and-Cultivation-Facility

[153] Canada NewsWire. Curaleaf announces opening of branded store in Wells, Maine. July 23, 2021. Accessed August 29, 2021. https://www.prnewswire.com/news-releases/curaleaf-announces-opening-of-branded-store-in-wells-maine-301339967.html

[154] Cision PR Newswire. Curaleaf Florida partnership with “Badges of Courage” assists low-income breast cancer patients. November 6, 2019. Accessed August 29, 2021. https://www.prnewswire.com/news-releases/curaleaf-florida-partnership-with-badges-of-courage-assists-low-income-breast-cancer-patients-300953244.html

[155] Curaleaf. In addition to the ribbon. 2021. Accessed October 1, 2021. https://curaleaf.com/blog/in-addition-to-the-ribbon

[156] Canada NewsWire. Curaleaf announces multi-faceted, month-long fundraising initiative to raise awareness for breast cancer. October 6, 2020. Accessed August 28, 2021. https://www.newswire.ca/news-releases/curaleaf-announces-multi-faceted-month-long-fundraising-initiative-to-raise-awareness-for-breast-cancer-860473749.html

[157] Leighton P. Cannabis company holds Ice Bucket Challenge in memory of Pete Frates. August 13, 2020. Accessed August 29, 2021. https://www.salemnews.com/news/local_news/cannabis-company-holds-ice-bucket-challenge-in-memory-of-pete-frates/article_2206ab40-9650-5819-9a7d-fe8569d27592.html

[158] PR Newswire. Curaleaf announces new branding for Illinois retail locations, opening of new Westmont store and contribution to IL social equity partners. April 5, 2021. Accessed August 29, 2021. https://www.prnewswire.com/news-releases/curaleaf-announces-new-branding-for-illinois-retail-locations-opening-of-new-westmont-store-and-contribution-to-il-social-equity-partners-301261842.html

[159] Innovative Industrial Properties. The leading provider of real estate capital for the regulated cannabis industry. Accessed October 4, 2021. https://innovativeindustrialproperties.com/

[160] Edwards L. NOTL’s Heritage Trail gets a boost from Canopy Growth. NiagraThisWeek.com. October 1, 2020. Accessed September 1, 2021. https://www.niagarathisweek.com/community-story/10213894-notl-s-heritage-trail-gets-a-boost-from-canopy-growth/

[161] Harford E. Tweed donates over $34,000 to Smiths Falls’ Station Theatre. January 11, 2018. Accessed September 1, 2021. https://www.insideottawavalley.com/news-story/8047771-tweed-donates-over-34-000-to-smiths-falls-station-theatre/

[162] Harford E. Tweed donates $10,000 to help Pearl Street fire victims. May 8, 2017. Accessed September 1, 2021. https://www.insideottawavalley.com/news-story/7293925-tweed-donates-10-000-to-help-pearl-street-fire-victims/

[163] TMX Money. Canopy Growth Corporation gives back to the communities it calls home. January 20, 2020. Accessed September 1, 2021. https://money.tmx.com/en/quote/WEED/news/4717662668294336

[164] Canopy Growth Corporation donates $25,000 to Smiths Falls food bank. *Smiths Falls Record News*. October 8, 2020. Accessed August 28, 2021. https://www.lexisnexis.com/en-us/professional/academic/nexis-uni.page

[165] Canopy Growth donates to homeless shelter. *Telegraph-Journal*. December 21, 2019. Accessed September 1, 2021. https://tj.news/daily-gleaner/101139260

[166] Grochowski S. Aldergrove food bank winter donations “blow away” volunteer managers. *Aldergrove Star*. January 15, 2020. Accessed September 2, 2021. https://www.aldergrovestar.com/home/aldergrove-food-bank-winter-donations-blow-away-volunteer-managers/

[167] Harford E. Tweed donates over $34,000, 2,500 pounds of food to Smiths Falls food bank. December 21, 2017. Accessed August 28, 2021. https://www.insideottawavalley.com/community-story/8019333-tweed-donates-over-34-000-2-500-pounds-of-food-to-smiths-falls-food-bank/

[168] Wilgosh A. 4/20 is donation day from local Tweed. April 20, 2021. Accessed August 28, 2021. https://www.thegraphicleader.com/news/local-news/4-20-is-donation-day-from-local-tweed

[169] Canada NewsWire. Spectrum Therapeutics announces support for Wounded Warriors Canada and its mental health services for veterans and first responders. October 28, 2020. Accessed September 5, 2021. https://www.newswire.ca/news-releases/spectrum-therapeutics-announces-support-for-wounded-warriors-canada-and-its-mental-health-services-for-veterans-and-first-responders-871931552.html

[170] Harford E. Local leaders help out on McHappy Day in Smiths Falls. May 2, 2018. Accessed August 29, 2021. https://www.insideottawavalley.com/community-story/8584048-local-leaders-help-out-on-mchappy-day-in-smiths-falls/

[171] Harford E. Smiths Falls & District Chamber of Commerce honours local businesses at AGM. November 29, 2017. Accessed September 1, 2021. https://www.insideottawavalley.com/news-story/7971016-smiths-falls-district-chamber-of-commerce-honours-local-businesses-at-agm/

[172] Market News Publishing. Canopy Growth calls New Brunswick home—company plans to create 136 high quality local jobs. July 5, 2018. Accessed September 1, 2021. https://markets.businessinsider.com/news/stocks/canopy-growth-calls-new-brunswick-home-company-plans-to-create-136-high-quality-local-jobs-1027344590

[173] Market News Publishing. Canopy Growth and New Brunswick sign historic supply MOU. September 15, 2017. Accessed September 1, 2021. https://www.lexisnexis.com/en-us/professional/academic/nexis-uni.page

[174] Parent Action on Drugs. About us. October 30, 2020. Accessed November 5, 2021. https://web.archive.org/web/20201030142536/http://parentactionondrugs.org/about-us/

[175] Bryant J. The Cannabis Industry’s plastic problem. October 30, 2020. Accessed September 30, 2021. https://www.healthline.com/health/cannabis-packaging

[176] Subramaniam V. Battling COVID-19: Telecoms company Bell to donate 1.5 million masks to healthcare and frontline workers. *National Post*. April 18, 2020. Accessed September 4, 2021. https://nationalpost.com/news/canada/battling-covid-19-telecoms-company-bell-to-donate-1-5-million-masks-to-healthcare-and-frontline-workers

[177] Staff. Cannabis companies step up to help fight COVID-19. Newstex Blogs Green Market Report. March 25, 2020. Accessed August 30, 2021. https://www.greenmarketreport.com/cannabis-companies-step-up-to-help-fight-covid-19/

[178] Hasse J. BioSteel sends $2M in products to aid hospitals, first responders and COVID-19 patients. April 14, 2020. Accessed August 30, 2021. https://finance.yahoo.com/news/biosteel-sends-2m-products-aid-164048819.html?fr=sycsrp_catchall

[179] GlobeNewswire. Green Thumb Industries reports first quarter 2021 results. May 12, 2021. Accessed September 3, 2021. https://www.globenewswire.com/news-release/2021/05/12/2228654/0/en/Green-Thumb-Industries-Reports-First-Quarter-2021-Results.html

[180] GlobeNewswire. Green Thumb Industries to open 49th retail location, Rise Monroeville in Pennsylvania, on October 21. October 20, 2020. Accessed September 1, 2021. https://www.globenewswire.com/news-release/2020/10/20/2110925/0/en/Green-Thumb-Industries-to-Open-49th-Retail-Location-Rise-Monroeville-in-Pennsylvania-on-October-21.html

[181] GlobeNewswire. Green Thumb Industries to open Rise Warminster in Pennsylvania, its 62nd retail location in the nation, on August 10. August 9, 2021. Accessed August 29, 2021. https://www.globenewswire.com/en/news-release/2021/08/09/2276940/0/en/Green-Thumb-Industries-to-Open-Rise-Warminster-in-Pennsylvania-Its-62nd-Retail-Location-in-the-Nation-on-August-10.html

[182] GlobeNewswire. Green Thumb Industries (GTI) supports Breast Cancer Awareness Month with limited edition RYTHM for a Cause vaporizer pens. September 27, 2018. Accessed September 3, 2021. https://www.globenewswire.com/news-release/2018/09/27/1577146/0/en/Green-Thumb-Industries-GTI-Supports-Breast-Cancer-Awareness-Month-with-Limited-Edition-RYTHM-for-a-Cause-Vaporizer-Pens.html

[183] GlobeNewswire. Green Thumb Industries’ Dogwalkers cannabis brand creates the “Bailey Legacy Fund” to support animal rescue organizations; makes donations to five nonprofits. January 28, 2021. Accessed August 30, 2021. https://www.globenewswire.com/news-release/2021/01/28/2165679/0/en/Green-Thumb-Industries-Dogwalkers-Cannabis-Brand-Creates-the-Bailey-Legacy-Fund-to-Support-Animal-Rescue-Organizations-Makes-Donations-to-Five-Nonprofits.html

[184] GlobeNewswire. Green Thumb Industries reports record results for the fourth quarter and full year 2020. March 17, 2021. Accessed August 28, 2021. https://www.globenewswire.com/news-release/2021/03/17/2194911/0/en/Green-Thumb-Industries-Reports-Record-Results-for-the-Fourth-Quarter-and-Full-Year-2020.html

[185] GlobeNewswire. Green Thumb Industries’ Dogwalkers cannabis brand partners with animal rescue organizations to raise awareness. December 12, 2018. Accessed September 3, 2021. https://www.globenewswire.com/news-release/2018/12/12/1665856/0/en/Green-Thumb-Industries-Dogwalkers-Cannabis-Brand-Partners-with-Animal-Rescue-Organizations-to-Raise-Awareness.html

[186] Hasse J. Green Thumb Industries relaunches “Dogwalkers” brand, with community giveback initiatives—and yes, a lot of pup love. July 24, 2019. Accessed September 3, 2021. https://www.benzinga.com/markets/cannabis/19/07/14120878/green-thumb-industries-relaunches-dogwalkers-brand-with-community-giveback-initiatives-and-yes-a

[187] GlobeNewswire. Green Thumb Industries to open Rise Salem in Virginia, its 60th retail location in the nation, on August 2. July 30, 2021. Accessed September 2, 2021. https://www.globenewswire.com/en/news-release/2021/07/30/2272037/0/en/Green-Thumb-Industries-to-Open-Rise-Salem-in-Virginia-Its-60th-Retail-Location-in-the-Nation-on-August-2.html

[188] Business Wire. Cresco Labs Employees Volunteer Over 5,000 Hours for Co’s 2020 “Make a Difference” initiative. January 18, 2021. Accessed September 2, 2021. https://www.businesswire.com/news/home/20210118005061/en/

[189] PR Newswire. Trulieve donates over 150 computers to local organizations across Florida. June 30, 2020. Accessed September 1, 2021. https://www.prnewswire.com/news-releases/trulieve-donates-over-150-computers-to-local-organizations-across-florida-301085453.html

[190] PR Newswire. GrowGeneration reports record Q1 2020 revenues of $33.0 million and record adjusted EBITDA of $2.7 million. May 14, 2020. Accessed August 30, 2021. https://www.prnewswire.com/news-releases/growgeneration-reports-record-q1-2020-revenues-of-33-0-million-and-record-adjusted-ebitda-of-2-7-million-301059046.html

[191] PR Newswire. GrowGeneration to donate hydroponic growing systems to urban farms and nonprofits across the US in partnership with Whole Cities Foundation. August 12, 2020. Accessed September 1, 2021. https://www.prnewswire.com/news-releases/growgeneration-to-donate-hydroponic-growing-systems-to-urban-farms-and-nonprofits-across-the-us-in-partnership-with-whole-cities-foundation-301109304.html

[192] PR Newswire. Spectrum Therapeutics partners with Canadian Mental Health Association’s Not Myself Today initiative. July 10, 2019. Accessed September 1, 2021. https://www.prnewswire.com/news-releases/spectrum-therapeutics-partners-with-canadian-mental-health-associations-not-myself-today-initiative-300882330.html

[193] Cresco Labs Highlights Its “Make a Difference” Initiative. Entertainment Close-Up. January 21, 2021. Accessed September 3, 2021. https://www.lexisnexis.com/en-us/professional/academic/nexis-uni.page

[194] PR Newswire. First Curaleaf-branded adult-use store opens in Maine. April 30, 2021. Accessed August 28, 2021. https://www.prnewswire.com/news-releases/first-curaleaf-branded-adult-use-store-opens-in-maine-301280980.html

[195] GlobeNewswire. Green Thumb Industries to open Rise Meadville in Pennsylvania, its 56th retail location in the nation, on March 31. March 26, 2021. Accessed September 1, 2021. https://www.globenewswire.com/news-release/2021/03/26/2199969/0/en/Green-Thumb-Industries-to-Open-Rise-Meadville-in-Pennsylvania-Its-56th-Retail-Location-in-the-Nation-on-March-31.html

[196] *New York Times* obituaries. Gerald Newman, 61, McDonald’s executive. October 15, 1992. Accessed November 8, 2021. https://www.nytimes.com/1992/10/15/obituaries/gerald-newman-61-mcdonald-s-executive.html

[197] The House Ronald McDonald Built. September 2014. Accessed November 8, 2021. https://www.qsrmagazine.com/charitable-giving/house-ronald-mcdonald-built

[198] Bloomberg. Media Advisory Canopy Growth to donate $100,000 to Forest Ontario to help plant millions of trees. August 15, 2019. Accessed August 29, 2021. https://www.bloomberg.com/press-releases/2019-08-15/media-advisory-canopy-growth-to-donate-100-000-to-forest-ontario-to-help-plant-millions-of-trees

[199] Hrebacka P. “Here’s to future growth”: Canopy Growth donates $100,000 to Forests Ontario in Kemptville. August 16, 2019. Accessed August 28, 2021. https://www.insideottawavalley.com/news-story/9553342--here-s-to-future-growth-canopy-growth-donates-100-000-to-forests-ontario-in-kemptville/

[200] PR Newswire. Curaleaf is ready to serve New Jersey’s growing market with new Bordentown location. August 25, 2021. Accessed September 1, 2021. https://www.prnewswire.com/news-releases/curaleaf-is-ready-to-serve-new-jerseys-growing-market-with-new-bordentown-location-301361928.html

[201] PR Newswire. Spectrum Therapeutics announces donation to support research and education initiatives led by the Canadian Sleep and Circadian Network. September 26, 2019. Accessed September 24, 2021. https://www.prnewswire.com/news-releases/spectrum-therapeutics-announces-donation-to-support-research-and-education-initiatives-led-by-the-canadian-sleep-and-circadian-network-300926073.html

[202] New Cannabis Ventures. Canopy Growth to fund professorship of cannabis science at University of British Coilumbia to fesearch [sic] the role of cannabis in addressing the opioid overdoes crisis. June 6, 2018. Accessed August 18, 2021. https://www.newcannabisventures.com/canopy-growth-to-fund-professorship-of-cannabis-science-at-university-of-british-columbia-to-research-the-role-of-cannabis-in-addressing-the-opioid-overdose-crisis/

[203] Columbia Care. Columbia Care launches “100,000,000 Ways to Break the Opioid Crisis” initiative. Columbia Care. Accessed September 28, 2021. https://web.archive.org/web/20211023011334/https://col-care.com/opioid-crisis/

[204] Canopy Growth. Santé Cannabis and Spectrum Therapeutics launch transformative medical cannabis training program for Québec healthcare professionals. September 16, 2019. Accessed November 9, 2021. https://www.canopygrowth.com/investors/news-releases/sante-cannabis-and-spectrum-therapeutics-launch-transformative-medical-cannabis-training-program-for-quebec-healthcare-professionals/

[205] Canopy Growth. Spectrum Therapeutics and Tree of Knowledge announce partnership to educate and support healthcare professionals. October 30, 2019. Accessed November 9, 2021. https://www.canopygrowth.com/investors/news-releases/spectrum-therapeutics-and-tree-of-knowledge-announce-partnership-to-educate-and-support-healthcare-professionals/

[206] Miller J. Number of Canadians buying legal medical marijuana triples in just one year. December 13, 2016. Accessed November 9, 2021. https://web.archive.org/web/20210225144637/https://ottawacitizen.com/news/local-news/number-of-canadians-buying-legal-medical-marijuana-triples-in-just-one-year

[207] Canadian AIDS Society. About us. April 20, 2016. Accessed November 8, 2021. https://www.cdnaids.ca/about-us/

[208] Cision. Global cannabis research leaders Beckley Foundation and Canopy Health Innovations partner to form Beckley Canopy Therapeutics. April 9, 2018. Accessed August 29, 2021. https://www.newswire.ca/news-releases/global-cannabis-research-leaders-beckley-foundation-and-canopy-health-innovations-partner-to-form-beckley-canopy-therapeutics-679115793.html

[209] Beckley Foundation. About the foundation. March 29, 2016. Accessed November 2, 2021. https://www.beckleyfoundation.org/about/the-foundation/

[210] Trulieve. TruVets. 2018-2021. Accessed September 28, 2021. https://www.trulieve.com/community-outreach/truvets

[211] Veterans Cannabis Project. About us. Accessed November 2, 2021. https://www.vetscp.org/about-us

[212] Curaleaf. Veterans Cannabis Project. 2021. Accessed September 28, 2021. https://curaleaf.com/veterans-cannabis-project/

[213] Cision PR Newswire. Curaleaf and the Veterans Cannabis Project launch national initiative to support efforts to increase veterans’ access to medical cannabis. March 28, 2019. Accessed August 30, 2021. https://www.prnewswire.com/news-releases/curaleaf-and-the-veterans-cannabis-project-launch-national-initiative-to-support-efforts-to-increase-veterans-access-to-medical-cannabis-300820282.html

[214] Veterans Cannabis Project. Partnerships. 2021. Accessed November 23, 2021. https://www.vetscp.org/partnerships

[215] Medical Cannabis Canada. Accessed September 29, 2021. https://patientaccess.ca/about-us/

[216] PR Newswire. Tweed and TerraCycle team up to take Cannabis packaging recycling program across Canada. April 22, 2019. Accessed September 2, 2021. https://www.prnewswire.com/news-releases/tweed-and-terracycle-team-up-to-take-cannabis-packaging-recycling-program-across-canada-300835543.html

[217] Agriculture XPRT. TerraCycle celebrate over one million pieces of cannabis packaging collected for recycling. October 10, 2019. Accessed August 29, 2021. https://www.agriculture-xprt.com/news/tweed-terracycle-celebrate-over-one-million-pieces-of-cannabis-packaging-collected-for-recycling-828233

[218] Canada NewsWire. Cannabis industry launches unique vape recycling program, partners with leading recycler. December 1, 2020. Accessed September 8, 2021. https://www.newswire.ca/news-releases/cannabis-industry-launches-unique-vape-recycling-program-partners-with-leading-recycler-880889592.html

[219] Rivera P. Canopy Growth partners with Parent Action on Drugs Canadian Students for Sensible Drug Policy to create youth-focused cannabis education initiatives. June 28, 2017. Accessed September 8, 2021. https://investingnews.com/daily/cannabis-investing/canopy-growth-partners-parent-action-drugs-canadian-students-sensible-drug-policy-create-youth-focused-cannabis-education-initiatives/

[220] Corporate social responsibility update from Canopy Growth Canadian Students for Sensible Drug Policy and Parent Action on Drugs. NCV Media LLC. Published April 4, 2018. Accessed September 7, 2021. https://www.newcannabisventures.com/corporate-social-responsibility-update-from-canopy-growth-canadian-students-for-sensible-drug-policy-and-parent-action-on-drugs/

[221] PR Newswire. Canopy Growth collaborates with Parent Action on Drugs to launch digital cannabis education tools. May 28, 2019. Accessed September 6, 2021. https://www.prnewswire.com/news-releases/canopy-growth-collaborates-with-parent-action-on-drugs-to-launch-digital-cannabis-education-tools-300857272.html

[222] Cresco Labs. Responsible advertising and marketing standards for the US cannabis industry. Fall 2020. Accessed September 8, 2021. https://www.crescolabs.com/wp-content/uploads/2020/10/Cresco_MarketingStandards_Fall20.pdf

[223] Sustainable Cannabis Coalition. About SCC. Accessed November 5, 2021. https://www.sustainablecannabiscoalition.com/about

[224] PR Newswire. Leading industry experts announce launch of Sustainable Cannabis Coalition. January 19, 2021. Accessed August 29, 2021. https://www.prnewswire.com/news-releases/leading-industry-experts-announce-launch-of-sustainable-cannabis-coalition-301210085.html

[225] Berke J, Lee JL. Insider Cannabis: sanity group’s $29 million pitch deck—Jay-Z’s investing in Black-owned cannabis startups—rest in peace, Keegan Peterson. *Business Insider*. January 22, 2021. Accessed September 1, 2021. https://africa.businessinsider.com/news/insider-cannabis-sanity-groups-dollar29-million-pitch-deck-jay-zs-investing-in-black/bf8rn3m

[226] Cronos Group. Responsibility. 2021. Accessed September 30, 2021. https://thecronosgroup.com/responsibility#this

[227] Cronos Group. Marketing code. Accessed September 8, 2021. https://thecronosgroup.com/CRONOS_MarketingCode_color.pdf

[228] PR Newswire. GrowGeneration announces Belushi’s farm partnership. April 30, 2021. Accessed August 30, 2021. https://www.prnewswire.com/news-releases/growgeneration-announces-belushis-farm-partnership-301281161.html

[229] University of Georgia Involvement Network. Students For Sensible Drug Policy. 2022. Accessed July 21, 2022. https://uga.campuslabs.com/engage/organization/ssdp

[230] Students for Sensible Drug Policy. US chapters by region. 2021. Accessed November 17, 2021. https://ssdp.org/chapters/us/

[231] Students for Sensible Drug Policy. Chapters. 2017-2021. Accessed November 5, 2021. https://ssdp.org/chapters/

[232] Helmer J. The environmental downside of cannabis cultivation. June 18, 2019. Accessed September 30, 2021. https://daily.jstor.org/the-environmental-downside-of-cannabis-cultivation/

[233] CTVNews. The fast-growing cannabis industry has a big pollution problem. March 21, 2021. Accessed September 30, 2021. https://www.ctvnews.ca/climate-and-environment/the-fast-growing-cannabis-industry-has-a-big-pollution-problem-1.5356116

[234] Smith J, Thompson S, Lee K. “Public enemy No. 1”: tobacco industry funding for the AIDS response. SAHARA J. 2016;13(1):41-52. doi:10.1080/17290376.2016.1164617 27023371 PMC5068916

[235] Smith EA, Thomson K, Offen N, Malone RE. “If you know you exist, it’s just marketing poison”: meanings of tobacco industry targeting in the lesbian, gay, bisexual, and transgender community. Am J Public Health. 2008;98(6):996-1003. doi:10.2105/AJPH.2007.118174 18445800 PMC2377293

[236] Yerger VB, Malone RE. African American leadership groups: smoking with the enemy. Tob Control. 2002;11(4):336-345. doi:10.1136/tc.11.4.336 12432159 PMC1747674

[237] McDaniel PA, Malone RE. Creating the “desired mindset”: Philip Morris’s efforts to improve its corporate image among women. Women Health. 2009;49(5):441-474. doi:10.1080/03630240903238800 19851947 PMC2791497

[238] Malyshev A, Ganley S, Malyshev A, Ganley S. The challenges of getting social equity right in the state-legal cannabis industry. Reuters. July 22, 2021. Accessed October 14, 2021. https://www.reuters.com/legal/litigation/challenges-getting-social-equity-right-state-legal-cannabis-industry-2021-07-22/

[239] Edwards K, Ewing L, Mulkey W. Social equity—or lack thereof—in the cannabis industry. June 10, 2020. Accessed October 14, 2021. https://www.simplifya.com/social-equity-or-lack-thereof-in-the-cannabis-industry/

[240] Stern R. Cannabis advocates rip Arizona’s draft rules for “social equity” licenses. May 14, 2021. Accessed October 14, 2021. https://www.phoenixnewtimes.com/marijuana/cannabis-advocates-rip-arizona-rules-for-social-equity-dispensary-licenses-11554200

[241] Young CA. State urged to form equity fund to address cannabis industry access. June 16, 2021. Accessed October 14, 2021. https://www.metrowestdailynews.com/story/business/2021/06/16/massachusetts-equity-fund-address-cannabis-industry-access/5294925001/

[242] Daniels M. Can cannabis social equity programs work? Palm Springs is setting one up. August 29, 2020. Accessed October 14, 2021. https://www.desertsun.com/story/money/business/2020/08/29/can-social-equity-programs-solve-weed-industrys-race-problem/5447582002/

[243] Burns J. Black entrepreneurs work to fix LA’s cannabis equity program, root out “sharecropper” investing. September 3, 2020. Accessed October 14, 2021. https://www.forbes.com/sites/janetwburns/2020/09/03/black-entrepreneurs-help-fix-la-cannabis-equity-sharecropper-agreements-documentary/

[244] Peipert T, Blood MR. Social equity in marijuana industry still largely pipe dream. April 30, 2021. Accessed October 14, 2021. https://www.denverpost.com/2021/04/30/marijuana-industry-social-equity/

[245] MacKenzie R, Collin J. “A preferred consultant and partner to the Royal Government, NGOs, and the community”: British American Tobacco’s access to policy-makers in Cambodia. Glob Public Health. 2017;12(4):432-448. doi:10.1080/17441692.2016.1170868 27079136 PMC5589517

[246] Apollonio D, Glantz SA. Tobacco industry research on nicotine replacement therapy: “If anyone is going to take away our business it should be us”. Am J Public Health. 2017;107(10):1636-1642. doi:10.2105/AJPH.2017.303935 28817320 PMC5599147

[247] Mamudu HM, Hammond R, Glantz SA. Project Cerberus: tobacco industry strategy to create an alternative to the Framework Convention on Tobacco Control. Am J Public Health. 2008;98(9):1630-1642. doi:10.2105/AJPH.2007.129478 18633079 PMC2509616

[248] Lee K, Carrillo Botero N, Novotny T. “Manage and mitigate punitive regulatory measures, enhance the corporate image, influence public policy”: industry efforts to shape understanding of tobacco-attributable deforestation. Global Health. 2016;12(1):55. doi:10.1186/s12992-016-0192-6 27650401 PMC5029076

[249] Saloojee Y, Dagli E. Tobacco industry tactics for resisting public policy on health. Bull World Health Organ. 2000;78(7):902-910. 10994263 PMC2560805

[250] Simpson C. Occupational health and safety in the cannabis industry. Ann Work Expo Health. 2020;64(7):677-678. doi:10.1093/annweh/wxaa068 32696046 PMC9769117

[251] Martyny JW, Serrano KA, Schaeffer JW, Van Dyke MV. Potential exposures associated with indoor marijuana growing operations. J Occup Environ Hyg. 2013;10(11):622-639. doi:10.1080/15459624.2013.831986 24116667

[252] Wiegand DM, Methner MM, Grimes GR, . Occupational exposure to secondhand cannabis smoke among law enforcement officers providing security at outdoor concert events. Ann Work Expo Health. 2020;64(7):705-714. doi:10.1093/annweh/wxaa025 32219297 PMC8593821

[253] Rotering TL, Lempert LK, Glantz SA. Emerging indoor air laws for onsite cannabis consumption businesses in the US. Am J Prev Med. 2021;61(6):e267-e278. doi:10.1016/j.amepre.2021.05.012 34400035

[254] Brener ND, Bohm MK, Jones CM, . Use of tobacco products, alcohol, and other substances among high school students during the COVID-19 pandemic—Adolescent Behaviors and Experiences Survey, United States, January-June 2021. MMWR Suppl. 2022;71(3):8-15. doi:10.15585/mmwr.su7103a2 35358166 PMC8979600

[255] Smith EA, McDaniel PA. Covering their butts: responses to the cigarette litter problem. Tob Control. 2011;20(2):100-106. doi:10.1136/tc.2010.036491 20966130 PMC3209806

[256] EcoWatch. Cigarette butts: the most littered item in the world. April 25, 2019. Accessed June 2, 2022. https://www.ecowatch.com/cigarette-butts-litter-2635534590.html

[257] Lee K, Oh WY, Kim N. Social media for socially responsible firms: analysis of Fortune 500’s Twitter profiles and their CSR/CSIR ratings. J Bus Ethics. 2013;118(4):791-806. doi:10.1007/s10551-013-1961-2

[258] Nair AS, Khan SUS. Senate Democrats roll out draft bill to legalize weed July 14, 2021. Accessed October 18, 2021. https://www.reuters.com/world/us/us-senate-democrats-release-discussion-draft-federally-legalize-cannabis-2021-07-14/

[259] Devine J. Pot reformers weigh in on Senate legalization plan. *LA Weekly*. September 3, 2021. Accessed October 19, 2021. https://www.laweekly.com/pot-reformers-weigh-in-on-senate-legalization-plan/

[260] Room R, Cisneros Örnberg J. Government monopoly as an instrument for public health and welfare: lessons for cannabis from experience with alcohol monopolies. Int J Drug Policy. 2019;74:223-228. doi:10.1016/j.drugpo.2019.10.008 31698164

[261] Herrick C. The post-2015 landscape: vested interests, corporate social responsibility and public health advocacy. Sociol Health Illn. 2016;38(7):1026-1042. doi:10.1111/1467-9566.12424 27037612

[262] National Cannabis Industry Association. Our mission and values. 2021. Accessed October 20, 2021. https://thecannabisindustry.org/about-us/mission-values/

[263] Bykowicz J. Cannabis goes corporate: lobbyists, unions seek to shape marijuana industry. *Wall Street Journal*. May 8, 2021. Accessed October 20, 2021. https://www.wsj.com/articles/cannabis-goes-corporate-lobbyists-unions-seek-to-shape-marijuana-industry-11620466202

[264] US Cannabis Council. About us. Accessed October 20, 2021. https://www.uscc.org/about-us

[265] Power M. Stoners cheered when Canada legalised cannabis: how did it go so wrong? April 5, 2020. Accessed November 18, 2021. https://www.theguardian.com/society/2020/apr/05/stoners-cheered-when-canada-legalised-cannabis-how-did-it-all-go-wrong

