# Supplementary material for: Content Analysis of the Corporate Social Responsibility Practices of 9 Major Cannabis Companies in Canada and the US
Source: JAMA Netw Open. 2022 Aug 23;5(8):e2228088. doi: 10.1001/jamanetworkopen.2022.28088 (PMC9399867; doi:10.1001/jamanetworkopen.2022.28088)
Supplement: Supplement. — eTable 1. Overview of CSR Activities Between 2012 and 2021 Regarding Medical Cannabis Access and Utility as a Treatment of the 9 Largest Multinational Cannabis Companies as of January 2021 eTable 2. Overview of General CSR Activity Categories and Charity Focuses Between 2012 and 2021 Among the 9 Largest Multinational Cannabis Companies as of January 2021 [file jamanetwopen-e2228088-s001.pdf]

# Supplementary Online Content

Wakefield T, Glantz SA, Apollonio DE. Content analysis of the corporate social responsibility practices of 9 major cannabis companies in Canada and the US. *JAMA Netw Open*. 2022;5(8):e2228088. doi:10.1001/jamanetworkopen.2022.28088

**eTable 1.** Overview of CSR Activities Between 2012 and 2021 Regarding Medical Cannabis Access and Utility as a Treatment of the 9 Largest Multinational Cannabis Companies as of January 2021

**eTable 2.** Overview of General CSR Activity Categories and Charity Focuses Between 2012 and 2021 Among the 9 Largest Multinational Cannabis Companies as of January 2021

This supplementary material has been provided by the authors to give readers additional information about their work.

© 2022 Wakefield T et al. *JAMA Network Open*.

| **eTable 1.** Overview of CSR Activities Between 2012 and 2021 Regarding Medical Cannabis Access and Utility as a Treatment of the 9 Largest Multinational Cannabis Companies as of January 2021 | | |
|---|---|---|
| | Medical Access | Medical Utility |
| Curaleaf | Veterans Cannabis Project. Curaleaf, [266] 2021 | No activities identified. |
| | Hosted medical card clinics for veterans. No Author, [136] 2021 | |
| Innovative Industrial | No activities identified. | No activities identified. |
| Canopy Growth | Medical cannabis patient survey. | Partnership with Canadian AIDS Society to fund development of Canadian guidelines. |
| | | $2.5 million to investigate and research opioid addiction treatment to university of British Columbia, mitigation via cannabis. |
| | | Beckley Canopy Therapeutics Formation. No Author, [178] 2018 |
| | | Spectrum partners with Canadian Mental Health Association’s Not Myself Today No Author, [124] 2019 |
| | | Continuing medical education for doctors. Canopy Growth, [174] 2019,<br>Canopy Growth, [175] 2019, Miller, [176] 2016 |
| | | Spectrum Therapeutics donation to Canadian Sleep and Circadian Network. No Author, [171] 2019 |
| Green Thumb Industries | No activities identified. | No activities identified. |
| Cresco Labs | No activities identified. | No activities identified. |
| Trulieve | Truvet program to connect veterans to doctors for medical access. Trulieve, [180] 2021 | No activities identified. |
| Cronos Group | No activities identified. | No activities identified. |
| GrowGeneration | No activities identified. | No activities identified. |
| Columbia Care | No activities identified. | 10,000 Ways to Break Opioid crisis. No Author, [173] nd |
| Source: Data collected by the authors.<br>* Superscripts in the table are citations linked to each instance of a CSR activity or theme. | | |

© 2022 Wakefield T et al. *JAMA Network Open*.

**eTable 2.** Overview of General CSR Activity Categories and Charity Focuses Between 2012 and 2021 Among the 9 Largest Multinational Cannabis Companies as of January 2021

| Company | CSR activity category | | | | | | | | | | | | | | | | | |
|---|---|---|---|---|---|---|---|---|---|---|---|---|---|---|---|---|---|---|
| | **Local causes** | **Food relief** | **Homelessness** | **Antipoverty** | **Veterans** | **Diversity** | **Health and medical care** | **Housing** | **Animals** | **Youth** | **Job training** | **Education** | **Sustainability** | **COVID-19** | **Miscellaneous** | **Donated portion of sales from limited-edition products** | **Donated proceeds of some store opening days** | **Brands dedicated to charitable donations** |
| Curaleaf Holdings Inc | NA | PR Newswire,[76] 2021; PR Newswire,[149] 2021; Canada NewsWire,[150] 2020; Canada NewsWire,[151] 2021 | Canada NewsWire, [150] 2020 | PR Newswire,[76] 2021; Curaleaf Holdings Inc,[152] 2021 | PR Newswire,[76] 2021; Canada NewsWire,[153] 2021 | NA | Cision PR Newswire,[154] 2019; Curaleaf Holdings Inc,[155] 2021; Canada NewsWire,[156] 2020 | Canada Newswire,[153] 2021; Leighton,[157] 2020 | NA | NA | PR Newswire,[158] 2021 | NA | NA | PR Newswire,[76] 2021 | NA | NA | NA | NA |
| Innovative Industrial Properties | Innovative Industrial Properties,[159] 2021 | Innovative Industrial Properties,[159] 2021 | NA | NA | NA | NA | Innovative Industrial Properties,[159] 2021 | NA | NA | Innovative Industrial Properties, [159] 2021 | NA | NA | Innovative Industrial Properties,[159] 2021 | NA | NA | NA | NA | NA |
| Canopy Growth Corporation | Edwards,[160] 2020; Harford,[161] 2018; Harford,[162] 2017; TMX Money,[163] 2020 | No author,[164] 2020; No Author,[165] 2019; Grochowski,[166] 2020; Harford,[167] 2017 | No author,[165] 2019; Wilgosh,[168] 2021 | NA | Canopy Growth Corporation,[79] ND; Canada NewsWire,[169] 2020 | NA | Harford,[170] 2018 | NA | NA | Harford, [171] 2017 | Market News Publishing,[172] 2018 | Market News Publishing,[173] 2017 | Parent Action on Drugs,[174] 2020; Bryant,[175] 2020 | Canopy Growth Corporation, [79] ND; Canada NewsWire,[169] 2020; National Post,[176] 2020; Staff,[177] 2020; Hasse,[178] 2020 | NA | NA | NA | NA |
| Green Thumb Industries Inc | NA | GlobeNewswire, [179] 2021; GlobeNewswire, [180] 2020 | GlobeNewswire, [181] 2021; GlobeNewswire, [179] 2021 | GlobeNewswire, [181] 2021 | GlobeNewswire, [179] 2021 | +GlobeNewswire, [179] 2021 | GlobeNewswire, [182] 2018; GlobeNewswire, [179] 2021 | GlobeNewswire, [179] 2021 | GlobeNewswire, [183] 2021; GlobeNewswire, [184] 2021; GlobeNewswire, [185] 2018; Hasse,[186] 2019 | NA | NA | NA | NA | NA | GlobeNewswire, [179] 2021 | GlobeNewswire, [182] 2018 | GlobeNewswire, [179] 2021; GlobeNewswire, [187] 2021 | GlobeNewswire, [183] 2021; GlobeNewswire, [184] 2021; GlobeNewswire, [185] 2018; Hasse,[186] 2019 |
| Cresco Labs Inc | NA | Business Wire,[188] 2021 | Business Wire,[188] 2021 | Business Wire,[188] 2021 | Business Wire,[188] 2021 | NA | Business Wire,[188] 2021 | NA | NA | NA | Businesswire,[100] 2020 | NA | Business Wire,[188] 2021 | Business Wire,[100] 2020 | Business Wire,[188] 2021 | NA | NA | NA |
| Trulieve | PR Newsline,[189] 2020 | NA | NA | NA | NA | NA | NA | NA | NA | NA | NA | NA | NA | Newswire,[189] 2020 | NA | NA | NA | NA |
| Cronos Group Inc | NA | NA | NA | NA | NA | NA | NA | NA | NA | NA | NA | NA | NA | NA | NA | NA | NA | NA |
| GrowGeneration Corp | NA | NA | NA | NA | NA | NA | NA | NA | NA | NA | NA | NA | NA | PR Newswire,[190] 2020 | PR Newswire,[191] 2020 | NA | NA | NA |
| Columbia Care | NA | NA | NA | NA | NA | NA | NA | NA | NA | NA | NA | NA | NA | NA | NA | NA | NA | NA |

Abbreviations: CSR, corporate social responsibility; NA, not applicable; ND, no date.

[a]Citations link to company involvement in CSR activity.

© 2022 Wakefield T et al. *JAMA Network Open*.
